# Single-molecule detection on a portable 3D-printed microscope

**DOI:** 10.1038/s41467-019-13617-0

**Published:** 2019-12-11

**Authors:** James W. P. Brown, Arnaud Bauer, Mark E Polinkovsky, Akshay Bhumkar, Dominic J. B. Hunter, Katharina Gaus, Emma Sierecki, Yann Gambin

**Affiliations:** 1 EMBL Australia Node in Single Molecule Science, and School of Medical Sciences, University of New South Wales, Sydney, 2052 NSW Australia; 2 The Institute for Molecular Bioscience, University of Queensland, St Lucia, QLD 4072 Australia; 3 ARC Centre of Excellence in Advanced Molecular Imaging, University of New South Wales, Sydney, 2052 NSW Australia

**Keywords:** Single-molecule biophysics, Prions, Protein aggregation, Education

## Abstract

Single-molecule assays have, by definition, the ultimate sensitivity and represent the next frontier in biological analysis and diagnostics. However, many of these powerful technologies require dedicated laboratories and trained personnel and have therefore remained research tools for specialists. Here, we present a single-molecule confocal system built from a 3D-printed scaffold, resulting in a compact, plug and play device called the AttoBright. This device performs single photon counting and fluorescence correlation spectroscopy (FCS) in a simple format and is widely applicable to the detection of single fluorophores, proteins, liposomes or bacteria. The power of single-molecule detection is demonstrated by detecting single α-synuclein amyloid fibrils, that are currently evaluated as biomarkers for Parkinson’s disease, with an improved sensitivity of >100,000-fold over bulk measurements.

## Abstract (web summary)

Single-molecule in vitro assays require dedicated confocal microscopes equipped with fluorescence correlation spectroscopy (FCS) modules. Here the authors present a compact, cheap and open-source 3D-printed confocal microscope for single photon counting and FCS measurements, and use it to detect α-synuclein aggregation.

## Introduction

A number of scientific instruments now possess single-molecule sensitivity and have enabled the discovery of novel disease biomarkers at extremely low concentrations^1^. Recent breakthroughs in single-molecule fluorescence super-resolution imaging have provided exquisite details into many biological processes in living cells^2–4^. In vitro, measurements of individual proteins have enabled the direct observation of folding trajectories or aggregation, leading to new understanding of fundamental molecular mechanisms^5^.

Despite being used extensively in biophysical laboratories, single-molecule fluorescence methods have remained a specialist field and are not yet widely used by biochemists or structural biologists. This could be owing to the fact that these single-molecule instruments are expensive, and extensive training is needed for the collection and analysis of high-quality data sets. For single-molecule fluorescence measurements in vitro, assays are performed on dedicated confocal microscopes equipped with fluorescence correlation spectroscopy (FCS) modules or on microscopes configured for total internal reflection fluorescence (TIRF). Recently, super-resolution imaging has spectacularly entered the field of cell biology^3,4^. Commercial setups are now more user-friendly and compact versions do not require dedicated dark rooms and classical air-suspended optical tables to minimise vibrations^6^. However, as discussed in a recent review, the financial barriers to buy and maintain a commercial turn-key microscope are still important for most laboratories^7^. Many groups are developing their own high-end instruments^8^, acquisition hardware, and software, but the blueprints of these setups are often too complex for replication by other groups.

Recent years have witnessed a community-driven effort toward creating simpler and cheaper instruments, especially for super-resolution imaging^9^, single-molecule FRET^10^ and smartphone fluorescence microscopy^11^. The first developments used commercially available optical elements and supports to assemble compact single-molecule setups without the need for the traditional microscope body^12^. The Hohlbein laboratory developed the MiCube^13^, a modular instrument to perform TIRF microscopy. The MiCube is assembled from 30 elements, either CNC milled from aluminium or commercially available (such as translation stages, mirror, and dichroic holders). This setup is made cost-efficient by using plastic, 3D-printed small elements and custom holders, without affecting performance.

**Fig. 1:**
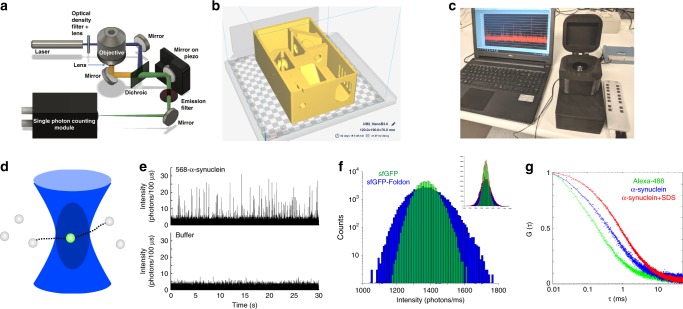
Design and characterisation of 3D-printed confocal microscope. **a** Overview of optical path. The design utilises a single excitation laser, which is focused to a very small (femtolitre) observation volume using a water-immersion objective (Zeiss × 40 or × 63, 1.2 NA). The emitted fluorescence is separated from the excitation fluorescence using a dichroic mirror and further filtered before detection by a single photon avalanche photodiode (SPAD, Micro Photon Devices). **b** 3D design of the housing for 3D printing; mirrors are pre-aligned within housing and filters, dichroic, and laser line can be easily exchanged. **c** Photograph of microscope compared with a laptop. **d** Principle of confocal detection of individual molecules freely floating in solution. **e** Detection of individual α-syn molecules labelled with Alexa-568 in water (top) compared with water alone (bottom). These measurements were performed on a normal wet bench, in broad daylight. 532 nm excitation, acquired at 10 kHz for 30 s. **f** Photon counting histograms (450 nm excitation, acquired at 1 kHz for 30 s) of sfGFP (green) and sfGFP-foldon (blue), an sfGFP-tagged trimerisation domain. **f** Data from main figure fit to Gaussian distribution for N&B analysis, see main text. **g** Fluorescence correlation spectroscopy (average of 8 × 10 s traces acquired at 1 MHz) of a fluorophore with a known diffusion coefficient (Alexa-488, 10 nm, green) and 10 nm Alexa-488 labelled α-syn in the absence (blue) and presence (red) of 10 mm sodium dodecyl sulphate (SDS) micelles.

Here, we go one step further and design an optical setup as a single 3D-printed plastic body to remove spatial limitations imposed by commercial mirrors, filters, or dichroic holders. We present a simplified single-molecule confocal system, the AttoBright, which requires minimal assembly with all optical components held in pre-aligned positions (Fig. 1a–c and Supplementary Fig. 1). The physical parameters of the AttoBright are characterised by FCS, and the instrument is used to perform single-molecule assays which detect single proteins, protein aggregates, liposomes, and bacteria. In particular, we detect individual α-synuclein (α-syn) amyloid fibrils using an amyloid-specific dye, Thioflavin T (ThT), with an improved sensitivity over bulk measurements of at least five orders of magnitude.

## Results

### AttoBright: compact low-cost single-molecule detection

To increase accessibility and reduce costs associated with single-molecule detection capabilities, we designed a simplified optical path that can be housed in a single 3D-printed block where all optical components are pre-aligned (Fig. 1a).

3D printing enables us to extend the concept of open access microscopes, as the exact reproduction of the setups is made possible by simply sharing the CAD files (Supplementary Software). The instrument can therefore be duplicated rapidly and with high fidelity, in laboratories or in classrooms. Unlike conventional optical setups or current homebuilt instruments, reproduction of the device does not require machining of custom elements in metal, expertise in optics, time-intensive alignment, or calibration. Furthermore, the laser, dichroic, and filters can easily be changed to optimise detection of the fluorophore of interest. The device self-aligns automatically; fine alignment is performed with a piezo motor connected to either a remote-control or an automated alignment algorithm (Supplementary Software). This device has a small footprint (10 cm × 20 cm), operates on a standard bench or desk, and is run from a conventional laptop. As the sample chamber and optics are encased during the measurement, the instrument can be used in ambient light with no requirement for a dedicated dark room. The instrument’s simple operation requires minimal training of users, as measurements can be performed by simply dropping a sample onto a coverslip. In the following sections we will refer to our device as the AttoBright.

To contribute to the community-driven development of new microscopes, we have included as supplementary information the details of this hardware (Supplementary Software, Methods) and software, including analysis packages and detailed codes for data acquisition, fitting and quantification (Supplementary Software).

### Single-molecule and fluorescence fluctuation experiments

Within a fixed experimental volume, individual molecules are detected as they diffuse in and out of the focal volume (Fig. 1d). Initial characterisation of the AttoBright was performed using well-established fluorescence fluctuation techniques: photon counting histograms (PCH)^14^, which extract the measured photon counts per molecule and the average number of molecules within the observation volume, moment analysis^15^ (also called Number and Brightness^16^), which is used to determine the degree of oligomerisation or aggregation of molecules, and FCS^17^, which reports on diffusion constants and molecular concentration.

The use of a high-end objective, a simplified optical path and optimised optical elements enables the high-performance required for single-molecule detection. Detection of individual protein molecules at concentrations below 100 pm was demonstrated using a range of concentrations of Alexa-568 labelled α-syn (Fig. 1e), and a global fit performed using PCH analysis (Supplementary Fig. 2, see methods for details of fit) determined the brightness of this reporter (*ε* = 277,600 cpsm). At higher concentrations, simple analysis of the first and second moments of the distribution of intensities^18^ allows the formation of small protein oligomers to be monitored. We performed experiments (Fig. 1f) on monomeric and trimeric green fluorescent protein (GFP). Monomeric superfolder GFP (sfGFP) was compared with a sfGFP-tagged foldon motif, a trimeric β-hairpin propeller derived from the C-terminal domain of T4 fibritin^19^. Both proteins were measured at concentrations of ~ 100 nm and PCHs calculated from 60 s traces revealing a broader distribution for sfGFP-foldon as compared with the sfGFP monomer, indicating oligomerisation. The B parameter $\left( {B = \frac{{\sigma ^2}}{\mu }} \right)$ contains contributions from molecular brightness and the diffusion coefficient of the molecule as well as shot noise, giving higher values for an increased brightness and/or physical size, and was calculated by fitting each histogram to a Gaussian distribution (Fig. 1f, inset), giving values of *B*_mono_ = 2.1 ± 0.1 and *B*_foldon_  = 5.7 ± 0.1.

### Characterisation of experimental volume by FCS

Protein interactions can also be monitored with FCS, which reports on the diffusion properties of the molecule of interest and changes in hydrodynamic radius. The technique is frequently used to determine the quality of the confocal excitation volume using a fluorescent molecule with a known diffusion coefficient^20^. From FCS measurements of Alexa-568 performed on the AttoBright configured with 532 nm excitation, the experimental volume was determined to be 0.96 ± 0.08 fL (mean ± standard deviation), with typical lateral radius ɷ_xy_ = 363 ± 6 nm and structure factor of 3.6 ± 0.3 (calculated from the average values across a range of Alexa-568 concentrations from 1.5–12 nm, Supplementary Fig. 3). The parameters of the focal volume are very similar to the data expected from a commercial setup calculated in the same manner (volume = 1.2 ± 0.15, ɷ_xy_ = 360 ± 20 nm, *a* = 4.6 ± 0.5)^20^. The focal volume and reproducibility of the data are the same across two AttoBright assemblies containing identical optical components (20 nm Alexa-568, volume = 0.96 ± 0.23 fL and 1.06 ± 0.23  fL, Supplementary Fig. 4). Instrument stability is also demonstrated by measurements of mean intensity, correlation amplitude and molecular brightness across multiple repeated measurements (Supplementary Fig. 5) as well as repeated FCS measurements on longer timescales (30 mins, Supplementary Fig. 6).

### Protein interactions by FCS: α-syn binding to SDS micelles

As the inexpensive and compact 532 nm laser diode modules have well-defined Gaussian beam profiles and the elliptical beam profiles of other lasers may increase the excitation volume (without a Gaussian-correcting optical fibre), we also tested the performance of the AttoBright with 405 nm and 450 nm laser modules. We determined whether we could monitor the binding of α-syn proteins to sodium dodecyl sulphate (SDS) micelles, an established membrane-mimetic^21^. Figure 1g shows autocorrelation curves of free Alexa-488 (green), Alexa-488 labelled α-syn (blue) and Alexa-488 labelled α-syn in the presence of an excess of SDS micelles (red). Calibration using free Alexa-488, which has a known diffusion coefficient^22^ of 414 μm^2^/s, shows a larger experimental volume (2.6 ± 0.4 fl with a lateral radius ɷ_xy_ = 614 ± 27 nm). Despite this, the diffusion coefficients of α-syn in the absence (167 ± 8 μm^2^/s) and presence of 10 mm SDS (78 ± 16 μm^2^/s) could be determined (Supplementary Fig. 7) and were similar to the literature values for α-syn monomer^23^ and SDS micelles^24^, respectively. Diffusion times (*τ*_D_) of repeated measurements were stable at concentrations of 1–100 nm (Supplementary Fig. 8). These compare to values of Alexa-568 labelled α-syn in the absence (183 ± 2 μm^2^/s) and presence of 10 mm SDS (85 ± 2 μm^2^/s) measured using the AttoBright configured with 532 nm excitation (Supplementary Fig. 9).

### Single particle detection of liposomes and bacteria

Various applications were tested in proof-of-concept experiments. We rationalised that if the system can detect single fluorophores, larger objects would be easily quantifiable. To this end, liposomes composed of a 50:50 mixture of 1-palmitoyl-2-oleoyl-glycero-3-phosphocholine (POPC) and 1-palmitoyl-2-oleoyl-sn-glycero-3-phospho-l-serine (POPS) containing Alexa-594 were measured using the 532 nm excitation configured AttoBright and their disruption monitored upon addition of a 1% Triton X-100 solution (Supplementary Fig. 10). This membrane permeabilisation measurement could be applied, for example, to the study of pore formation. Further, larger objects such as bacteria can be easily detected at the single particle level. To demonstrate this, we swabbed plates contaminated by bacteria expressing mCherry, recovered bacteria from the swabs and recorded large peaks of intensity corresponding to single bacteria (Supplementary Figs 11 and 12). Using fluorescent antibodies, rapid detection of specific bacteria should be feasible without overnight growth and amplification. This would find applications in microbiology and food safety for the detection of pathogens.

### Detection of individual α-syn amyloid fibrils

The major advantage of single-molecule assays is the ability to detect rare species in complex samples and reveal features, which are hidden by ensemble averaging. We rationalised that this would be especially advantageous if the target proteins exist in monomeric and different aggregated forms, as in the case of misfolding and pathological aggregation of proteins. In many neurodegenerative diseases, proteins misfold and eventually form large amyloid deposits, and the study of the oligomerisation and amyloidogenesis of these proteins (which include amyloid-β, Tau, TDP-43, and α-syn, among others) is a major research area^25^.

**Fig. 2:**
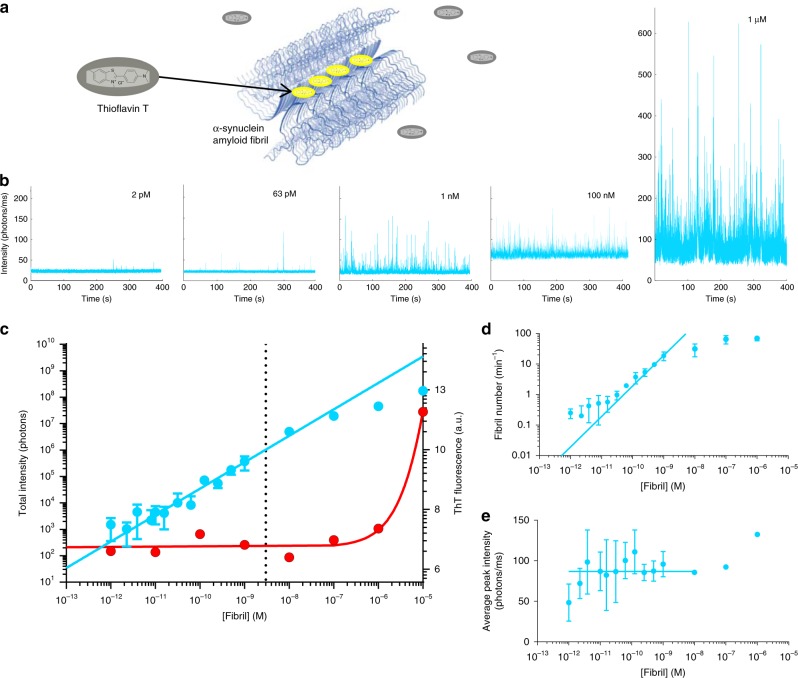
Application to the detection of protein aggregates. **a** ThT binds specifically to amyloid fibrils and increases fluorescence upon binding. **b** Example traces of ThT (1 μm) binding to amyloid fibrils and measurement of different concentrations of mature α-syn amyloid fibrils (405 nm excitation, acquired at 100 Hz for 400 s). **c** Total ThT intensity detected by AttoBright (light blue) or a bulk measurement using a fluorescence plate reader (BMG Labtech, red). For concentrations below 3 nm (dotted line), the AttoBright data are analysed by integrating individual peaks. For concentrations above 3 nm, the AttoBright data are analysed by integrating total signal intensity. The dotted line marks concentration at which individual fluorescent bursts cannot be reliably separated. The dotted line is three orders of magnitude lower than the typical sensitivity of a bulk measurement. **d**, **e** Deconvolution of number and size of aggregates measured in **c**. **d** Number of ThT-positive species detected by AttoBright across serial fibril dilutions. Linear range ~ 10^−11^–10^−9^ m. **e** Average peak intensity remains constant with fibril length across a wide range of concentrations (~ 10^−11^–10^-8^ m). Error bars are mean ± s.d of 3 × 400 s measurements.

The formation of amyloid over time is monitored using ThT, a dye widely used for biophysical assays and histological staining, owing to the increase in fluorescence upon binding to the cross-β-sheet structure of amyloid aggregates (Fig. 2a). Direct detection of low concentrations of ThT-positive aggregates was made possible by single-molecule imaging by TIRF^26^. We set out to use the AttoBright to characterise the sensitivity of single aggregate ThT detection, in which multiple ThT-molecules bind to each aggregate, compared with ensemble bulk measurements.

The detection limit for α-syn aggregates was determined using isolated amyloid fibrils formed from recombinantly produced and purified α-syn. A sample containing a known concentration (10 μm monomer equivalent) of α-syn fibrils was sequentially diluted into a ThT-containing solution and measured using both the AttoBright and a fluorescence plate reader (Fluostar Omega, BMG Labtech). When bulk ThT intensity becomes indistinguishable from background (~ 1 µM), the single-molecule traces still show a very high number of peaks over average ThT fluorescence (Fig. 2b). The lowest concentration for which aggregates could be distinguished from background was 1 pM monomer equivalent (15 pg/ml), which corresponds to 150 fg in a typical 10 µl sample volume. As shown in Fig. 2c, direct counting of individual aggregates achieves an increase in sensitivity of at least five orders of magnitude compared with conventional bulk methods. The sensitivity of our simple benchtop instrument even compares favourably to more complex amyloid detection methods (10 pm by single-molecule TIRF imaging^26^ and another confocal setup^27^ and 40 pm using immunogold labelling and dark field microscopy^28^). A key advantage of the direct detection of individual diffusing particles is also the deconvolution of the number and size of aggregates, which is crucial in the context of disease, as a number of studies have linked smaller aggregated species to an increase in cellular toxicity^29–31^. In the absence of other factors such as fibril packing^32^, the fluorescence intensity of each ThT burst reports on the relative length of individual fibrillar aggregates owing to the increase in exposed ThT binding sites per particle. Both the number (Fig. 2d) and information on the relative size (Fig. 2e) of the aggregates can be evaluated from the raw traces. As expected from a titration, the overall increase in signal we detected resulted from an increase in the number of species of similar sizes. Below 10 pm, there is no longer a linear relationship between fibril dilutions and their number and size, with the number of aggregates higher than expected and their size lower despite total intensity remaining linear. This is possibly owing to the reduced stability of aggregates at these concentrations, leading to their fragmentation.

The direct detection of number and relative size of protein fibrils can give rapid information on the aggregation pathway, distinguishing between the elongation and nucleation phases in amyloid formation.

## Discussion

We have designed and built a simplified single-molecule confocal system and demonstrated various experimental applications, including protein:micelle interactions, liposome disruption, bacterial detection, and pathological protein aggregate detection. The latter has a sensitivity to 15 pg/ml, 10^6^-fold more-sensitive than bulk detection and similar to more complex single-molecule systems. The low-cost device self-aligns quickly and acquisition of photon counting, brightness or FCS measurements can be performed after minimal training. The open-source hardware and software for data acquisition and analysis will hopefully make single-molecule methods more accessible to non-specialists. Super-resolution imaging techniques have currently a dramatic impact in biology, and we hope that single-molecule spectroscopy will have a similar role to play for academic laboratories working at the protein level, in industrial settings or for diagnostic purposes, and in classrooms to expose students to new methods.

## Methods

### Protein, liposome, and fibril preparation

Wild-type and A90C α-syn in T7-7 vectors were expressed and purified according to previously described protocols^33,34^, divided into aliquots, flash frozen in liquid N_2_ and stored in 20 mm phosphate buffer (PB, pH 7.4, 0.01% NaN_3_) at − 80 °C. A90C α-syn was labelled with maleimide-modified Alexa Fluor 488 or Alexa Fluor 568 dyes (ThermoFisher Scientific, Waltham, MA, USA) via the cysteine thiol moiety. The labelled protein was purified from the excess of free dye by dialysis against PB at 4 °C overnight, divided into aliquots, flash frozen in liquid N_2_ and stored at − 80 °C.

Preformed fibrils, used to determine the detection limit for amyloid aggregates, were formed by incubating 200 μm monomeric α-syn (PBS, pH 7.4) at 45 °C with stirring by a Teflon bar. At 24 h intervals, the fibril solution was sonicated using a water bath sonicator for 15 mins. After 72 h, the fibril solutions were divided into 50 μm aliquots, flash frozen with liquid N_2_ and stored at − 20 °C until required. For the detection of fibrils experiments, the solutions were diluted to 5 μm in PBS and sonicated for a further 10 min just before use.

POPS and POPC lipids (Avanti Polar Lipids, Alabama, USA) dissolved in chloroform were dried with nitrogen gas and desiccated overnight. Lipid films were resuspended to a final concentration of 1 mg/ml in 10 mm Tris-HCl pH 7.4, 200 mm NaCl, 50 μm A594 and incubated for 15 min at 37 °C prior to extrusion with 100 nm polycarbonate membrane (Avanti Polar Lipids) to form unilamellar vesicles. Excess dye was removed by Sephadex G25 gravity column.

### Cell-free protein expression

*Leishmania tarentolae* extracts (LTE) were produced as previously described in detail.^35,36^ Plasmids containing Foldon in a cell-free expression vector (pCellFree_G10^37^), which contains a C-terminal sfGFP 8xHis tag, as well as the sfGFP-containing vector itself, were transcribed and translated in LTE (at a final DNA concentration of 20 nm). The reactions were incubated at 27 °C for 2 h and experiments were performed immediately after expression.

### 3D-printed housing

The simplified single-molecule confocal system presented here (Supplementary Fig. 1) is designed to be compact in size with a small number of optical elements, to reduce issues with misalignment and chromatic aberration and increase ease of use/assembly. To this end, a diverging lens fixed in the housing is used for transferring the light beam emitted by the light source to the sample, which shortens the optical path compared with using a converging lens with a pinhole. In addition, an achromatic doublet lens is used immediately prior to the objective meaning a single focusing element is used in the excitation and detection pathways. In the detection path, the 50 μm diameter active area of the single-photon APD is used as a pinhole, further reducing the number of optical elements required. Aligning the pinhole is performed by adjusting the mirror mounted on piezo-electric motors. This scanning mirror in a feedback arrangement is used to direct emitted light onto the active area of the detector in the *x*-y dimensions.

The 3D-printed housing was designed using FreeCAD software (version 0.16, *freecadweb.org) and* printed on an Ultimaker 2 + using the PLA plastic with a 0.4 mm nozzle. The printer setting were: layer height – 0.1 mm, wall thickness – 1.05 mm, top/bottom thickness – 0.8 mm, infill density 20% with 10% overlap percentage, print speed – 50 mm/s and travel speed 120 mm/s. The print was supported via a brim stuck to adhesion plate with a width of 8.0 mm.

### Single-molecule instrumentation

Several instruments, based on the same 3D-printed housing, were utilised for the acquisition of the data presented in this paper.

Instrument 1, for the acquisition of data using Thioflavin T, was built as follows. An elliptical beam, produced by a collimated laser diode, at a wavelength of 405 nm (CPS-405, ThorLabs) was first attenuated by a neutral density filter (1.0 density) before being expanded using a bi-concave lens. A dichroic beamsplitter (Di02-R488-25 × 36, Semrock) reflected the laser light and directed it, via a mirror, through a visible achromatic doublet lens to collimate the beam, which then entered into the back aperture of a × 40/1.15NA water-immersion objective (UAPON340, Olympus). The objective then focused the illumination beam to a diffraction-limited confocal spot within the sample. The emitted fluorescence was collected by the same objective, focused through the doublet lens and passed through the dichroic, before being directed, via a mirror set in a piezo directed optical mount (AG-M100N, Newport), through a 525/50 nm band-pass filter (FF03-525/50-25, Semrock) and directed via a final mirror onto the avalanche photodiode (APD) detector (50 μm diameter active area, PD-050-CTC, Micro Photon Devices). Outputs from the APD are connected to a USB data acquisition card (USB-CTR04, Measurement Computing), which counts the signal and combines them into time-bins, which are selected according to the application requirements (FCS: 1–10 μs, PCH: 10 μs, Brightness: 10 μs–1 ms, aggregate detection: 10 ms). Data acquisition was performed at room temperature, using custom-made acquisition software written in the LabView (National Instruments) programming environment. Laser power is ~ 450 μW.

Instrument 2, for the acquisition of data using GFP or the Alexa-488 fluorophore, is similar to the one described above except that the 405 nm laser diode is replaced with one producing an elliptical beam at a wavelength of 450 nm (CPS-450, Thorlabs). Laser power is ~ 450 μW.

Instrument 3, for the acquisition of data using the Alexa-568 fluorophore, was built as above, with the laser diode replaced with one producing a round beam at a wavelength of 532 nm (CPS-532, Thorlabs). The dichroic was replaced with a 561 nm dichroic beamsplitter (Di03-R561-t1–25 × 36, Semrock) and the emission filter with a 568 nm long pass filter (BLP01-568R-25, Semrock). The objective was replaced with a × 63/1.2 NA water-immersion objective (C-Apochromat, Zeiss). Laser power is ~ 450 μW.

### Single-molecule data acquisition hardware and software

The USB-CTR04 data acquisition card, controlled by a LabVIEW software routine, is used to record data from the APD detector in the AttoBright. The counter on the card has a maximum data input frequency of 48 MHz, faster than the detection frequency of the APD (including dead-time). When detecting a photon, the APD sends a high pulse to the acquisition card, and the counter on the card tallies the number of pulses, or detected photons. At the end of each time bin (i.e., 1 ms for a 1 kHz acquisition), the total number of pulses is recorded by the LabVIEW programme in a designated data file. The LabVIEW programme is used to control the acquisition frequency, and operates reliably at acquisition frequencies up to 1 MHz in short bursts (< 10 s) for FCS measurements, but is more commonly run at 1 kHz for longer periods to perform single-molecule brightness analysis. The programme also allows the user to set the total acquisition time per data file, and to record multiple files (to reduce individual file size for easier parsing). Finally, the programme displays a trace of the current signal being recorded, as well as the mean signal in the current data file (updated every second).

Another LabVIEW routine is used to align the laser excitation by scanning the piezo-actuated mirror through a given range in both axes, and measuring the detected signal at each step. After several rounds of decreasing the movement range and increasing precision, the laser is optimally aligned to create the detection volume at the focus of the optical objective.

Other acquisition and alignment modalities on the AttoBright are combinations or variations of these routines, optimised for different conditions.

### Single-molecule data analysis

FCS experiments were performed on instruments 2 and 3. Data were autocorrelated and fit to a one component diffusion model with triplet state correction, assuming a Gaussian detection volume:

$$G\left( \tau \right) = 1 + G(0)\left( {\frac{{\tau _{\mathrm{p}}}}{{1 \, - \, \tau _{\mathrm{p}}}}} \right)e^{ - \frac{\tau }{{\tau _{\mathrm{e}}}}}\frac{1}{{\left( {1 \, + \, \left( {\frac{\tau }{{\tau _{\mathrm{D}}}}} \right)} \right) \ast \left( {1 \, + \, a^{ - 2} \ast \left( {\frac{\tau }{{\tau _{\mathrm{D}}}}} \right)} \right)^{\frac{1}{2}}}} + G(\infty )$$

where *G*(*τ*) is the autocorrelated data, *G*(0)is the correlation at *τ* = 0, *τ*_p_ is the fraction of fluorophore in the triplet state, *τ*_e_ is the corresponding triplet state relaxation time, *τ*_D_ is the characteristic diffusion time of the fluorescent species, *a* is the structure factor (*z*_0_/*ω*_0_), where *z*_0_ and *ω*_0_ are the *e*^−2^ radii in the lateral and perpendicular direction relative to the optic axis, respectively, and *G*(∞) is the value of the autocorrelation as *τ* → ∞.

Autocorrelation and fits were performed using a custom Matlab routine utilising a nonlinear least squares fitting algorithm.

Effective volume was calculated from fit using $V_{eff} = \pi ^{\frac{3}{2}}{\omega _0}^3a$

Apparent brightness (*B*) experiments were performed on instrument 2. The B parameter was calculated using $B = \frac{{\sigma ^2}}{\mu }$ .

PCH experiments were performed on instrument 3. Data were fit to a 3D Gaussian PCH model using the Globals Unlimited software package (Laboratory for Fluorescence Dynamics, University of Illinois at Urbana-Champaign).

Fibril detection and protein aggregation experiments were performed on instrument 1. Data were analysed using a custom Matlab routine, which enabled quantification of ThT-positive species via an automated peak picking algorithm, which would count, integrate, and find the maximum intensity of each ThT-positive burst. Thresholds were set as mean +5 standard deviations of ThT alone up until concentrations where individual fluorescent bursts could not be reliably distinguished (corresponding to a fibril concentration of ~ 10 nm) at which point the total signal was integrated to give an ensemble measure of fibril concentration.

Matlab routines which perform FCS fitting, a simple brightness analysis and peak picking are included as supplementary software. In addition, a GUI (AttoBright Analyser), which operates as either a Matlab app or on Matlab Runtime (freely available) will also perform these functions and is included as supplementary software.

### Bacterial detection on the AttoBright

For bacterial detection using the AttoBright, *Escherichia coli* BL-21(AI) cells, transformed with an mCherry—mCherry expressing vector (pCellFree_G05^37^), were grown in LB + Ampicillin (100 μg/mL final concentration) with protein production induced by adding 0.2% Arabinose to the culture. The mCherry—mCherry tandem expressing cells were diluted to an O.D. of 0.08 and 200 μL of this solution was added to LB agar plates containing 100 μg/mL of Ampicillin. The cells were allowed to air dry for 2 min and a part of the plate was swabbed using a wet swab (cotton applicator wooden stick with single tip dipped in MilliQ water). The bacteria were retrieved by slow centrifugation (500 g for 1 min) and 10 μL sample was read for 120 s on the AttoBright (instrument 3). Data were analysed using a custom Matlab routine which enabled quantification of each fluorescent event via an automated peak picking algorithm. This counts, integrates, and finds the maximum intensity of each fluorescent event with a threshold set at mean + 5 standard deviations.

To study the bacterial detection efficacy of the AttoBright, mCherry—mCherry tandem expressing *E. coli* BL-21 (AI) cells (grown and induced as described above), were harvested and diluted to an O.D. of 0.2 and 20 μL of this sample was read for 60 s on the AttoBright (instrument 3). The bacterial solution was subsequently diluted twofold and each sample was read for 60 s on the AttoBright. Data were analysed as described above. To measure the O.D. of the samples, 1 mL of each sample was loaded in 1.5 mL semi-micro disposable cuvettes and the samples were read on a Biowave CO8000 cell density metre, using 1 mL LB as a blank measurement.

### Reporting summary

Further information on research design is available in the Nature Research Reporting Summary linked to this article.

## Supplementary information

Supplementary Information

Description of Additional Supplementary Files

Supplementary Software

Reporting Summary

## Data Availability

The Supplementary Software file contains the 3D printing STL files, LabView code and a GUI for data acquisition, Matlab code and a GUI for data analysis as well as a parts list and user guide for assembly/acquisition and analysis. All custom code is also available at https://gambinsiereckilab.github.io/AttoBright/

## Data availability

All the data supporting the findings of this study are available from the corresponding authors upon reasonable request.
