# Supplementary material for: Single-molecule detection on a portable 3D-printed microscope
Source: Nat Commun. 2019 Dec 11;10:5662. doi: 10.1038/s41467-019-13617-0 (PMC6906517; doi:10.1038/s41467-019-13617-0)
Supplement: Supplementary file 1 — Supplementary Information [file 41467_2019_13617_MOESM1_ESM.pdf]

# Single-molecule detection on a portable 3D-printed microscope

James W.P. Brown[1]*, Arnaud Bauer[1]*, Mark E Polinkovsky[1], Akshay Bhumkar[1], Dominic J.B. Hunter[2],Katharina Gaus[1,3], Emma Sierecki[1+] & Yann Gambin[1+]

1. EMBL Australia Node in Single Molecule Sciences, and School of Medical Sciences, Faculty of Medicine, University of New South Wales, Sydney Australia
2. The Institute for Molecular Bioscience, University of Queensland, St Lucia, QLD, 4072, Australia
3. ARC Centre of Excellence in Advanced Molecular Imaging, University of New South Wales, Sydney Australia

Correspondence should be sent to e.sierecki@unsw.edu.au, y.gambin@unsw.edu.au

# Supplementary Information

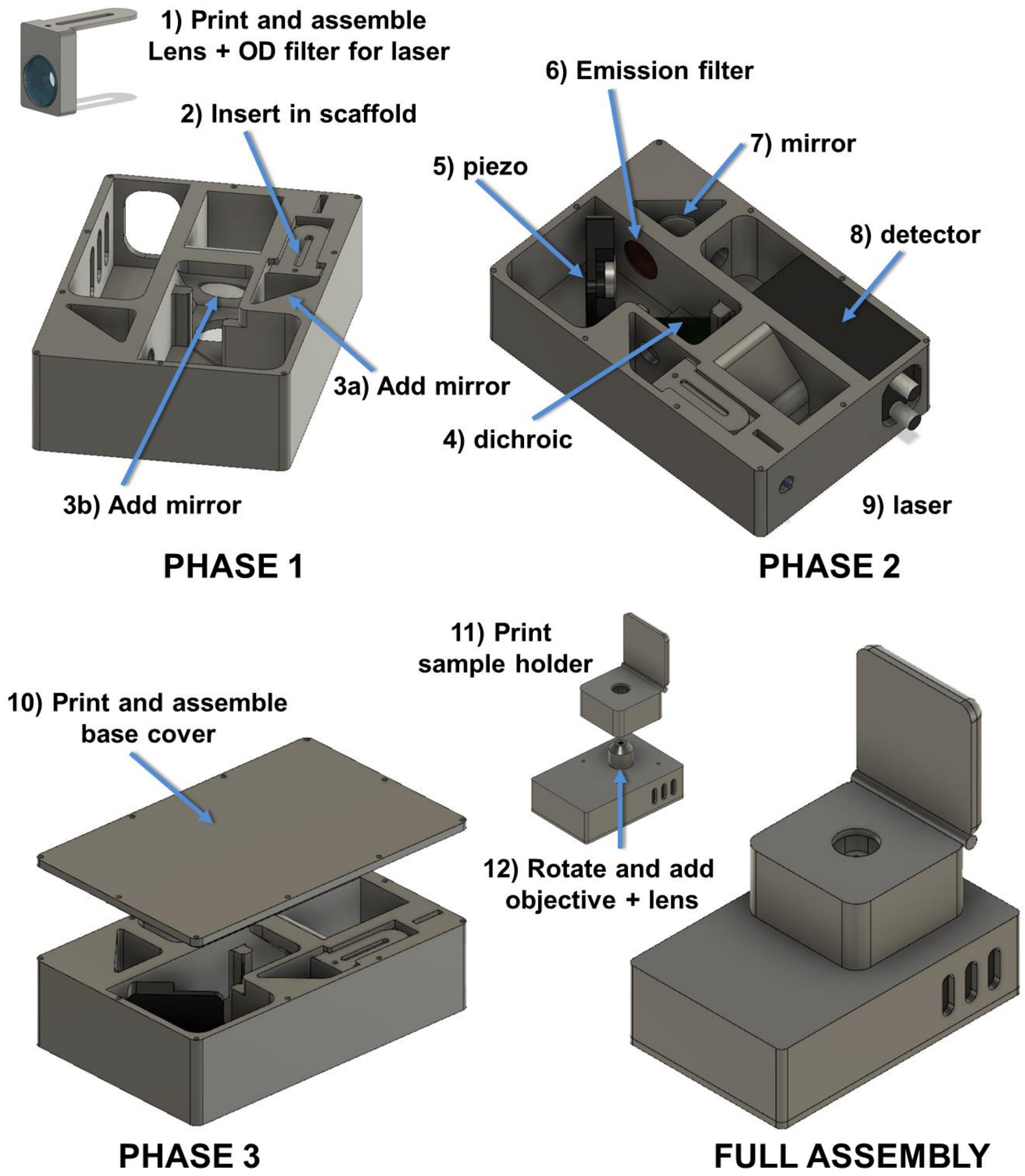


**Supplementary Figure 1: rapid description of the assembly steps**

0) Print the main scaffold (named "Printing_AttoBright body").
1) Print the holder for the diverging lens and the OD filter (named "Printing_Lens focus"). Assemble by inserting gently the OD filter in the inside of the holder, and the diverging lens on the outer face of the holder.
2) Insert in the AttoBright body and tighten loosely in place using a 4g x 12mm screw.
3) Add mirrors in the 45° holders and tighten in place through the side holes using SS3M6 screws, during assembly, mirrors may be held using kimwipe or similar.
4) Insert the dichroic by gentle vertical pressure; the plastic will hold the dichroic in place, for replacement of dichroic, instrument may need to be placed at ~40°C for 5 mins.
5) Insert the piezo and tighten firmly in place using a 4g x 12mm screw.
6) Insert the emission filter and tighten in place using SS3M6 screw.

7) Insert the mirror and tighten in place using SS3M6 screw.
8) Insert the detector; the detector will be held in place by tightening the base cover.
9) Insert the laser and tighten in place using SS3M6 screw.
10) Print the base cover (named “Printing_AttoBright base”) and screw onto AttoBright scaffold using a 4g x 12mm screw.
11) Print the sample holder (named “Printing_AttoBright sample holder”).
12) Flip the assembly and insert the lens and microscope objective.
13) Insert the sample holder on top of the AttoBright, and adjust height of the objective.

A full part list for 450 nm excitation AttoBright is available at https://gambinsiereckilab.github.io/AttoBright/.

Other instruments can be built using either the part numbers in the methods, or custom combinations of laser source, dichroic and emission filter according to requirements.

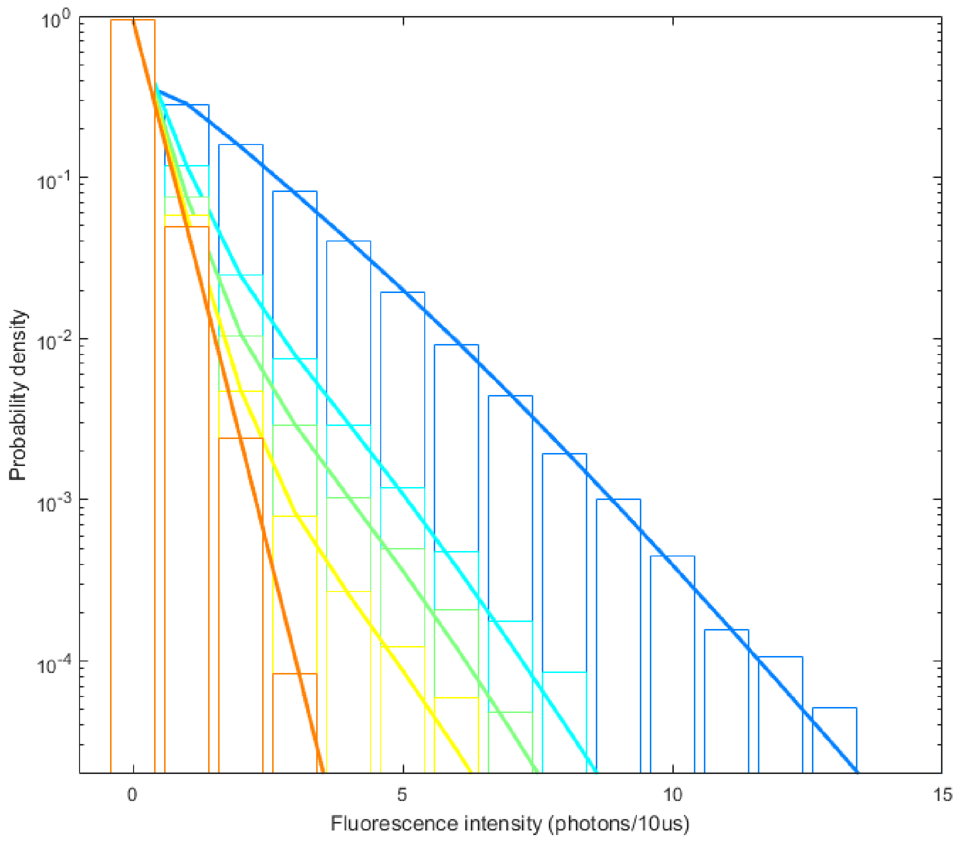


**Supplementary Figure 2** Photon counting histograms of Alexa-568 labelled α-synuclein at varying concentrations (from highest to lowest; blue, teal, green and yellow) and background (water, orange). A global fit was performed (see methods) using the Globals Unlimited software package (Laboratory for Fluorescence Dynamics, University of Illinois at Urbana-Champaign, Urbana, IL) on the multiple 568-α-synuclein concentrations to determine the brightness, ε, of this species, found to be 277,600 cpspm.

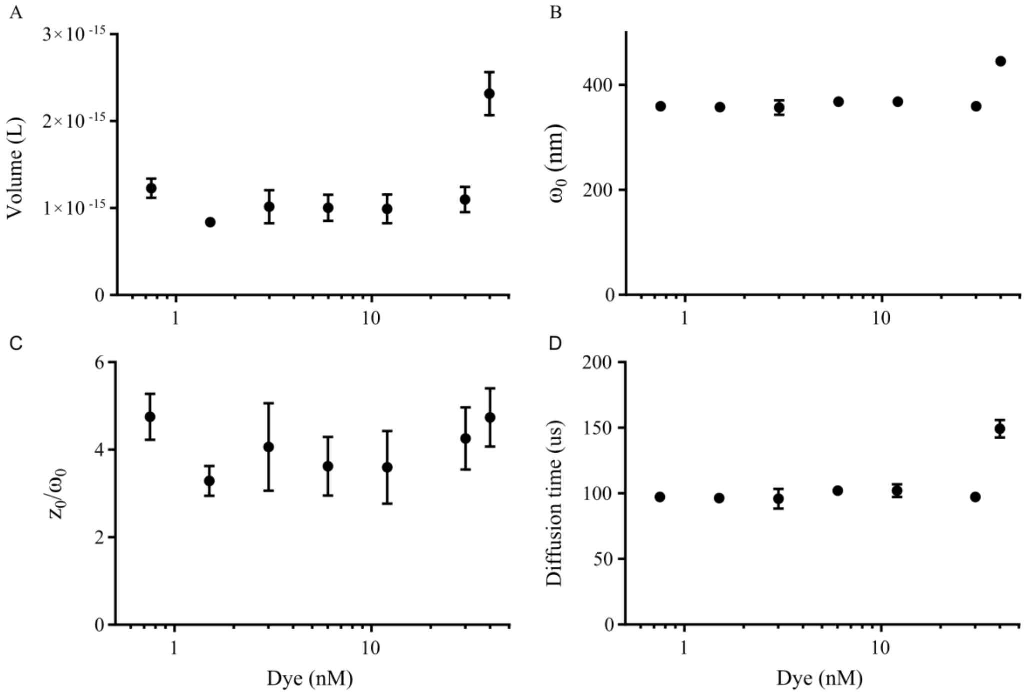


**Supplementary Figure 3 Physical parameters of confocal volume across a range of free Alexa-568 concentrations from 0.75 to 40 nM.**

(A) Confocal volume, L, from fit of autocorrelation data to the autocorrelation function for pure diffusion in a 3D confocal Gaussian volume (B) Lateral radius, $\omega_0$ (C) Structure factor, $a = \frac{z_0}{\omega_0}$ (D) Diffusion time $\tau_D$ in µs. Error bars are mean +/- s.d of 10 repeated measurements

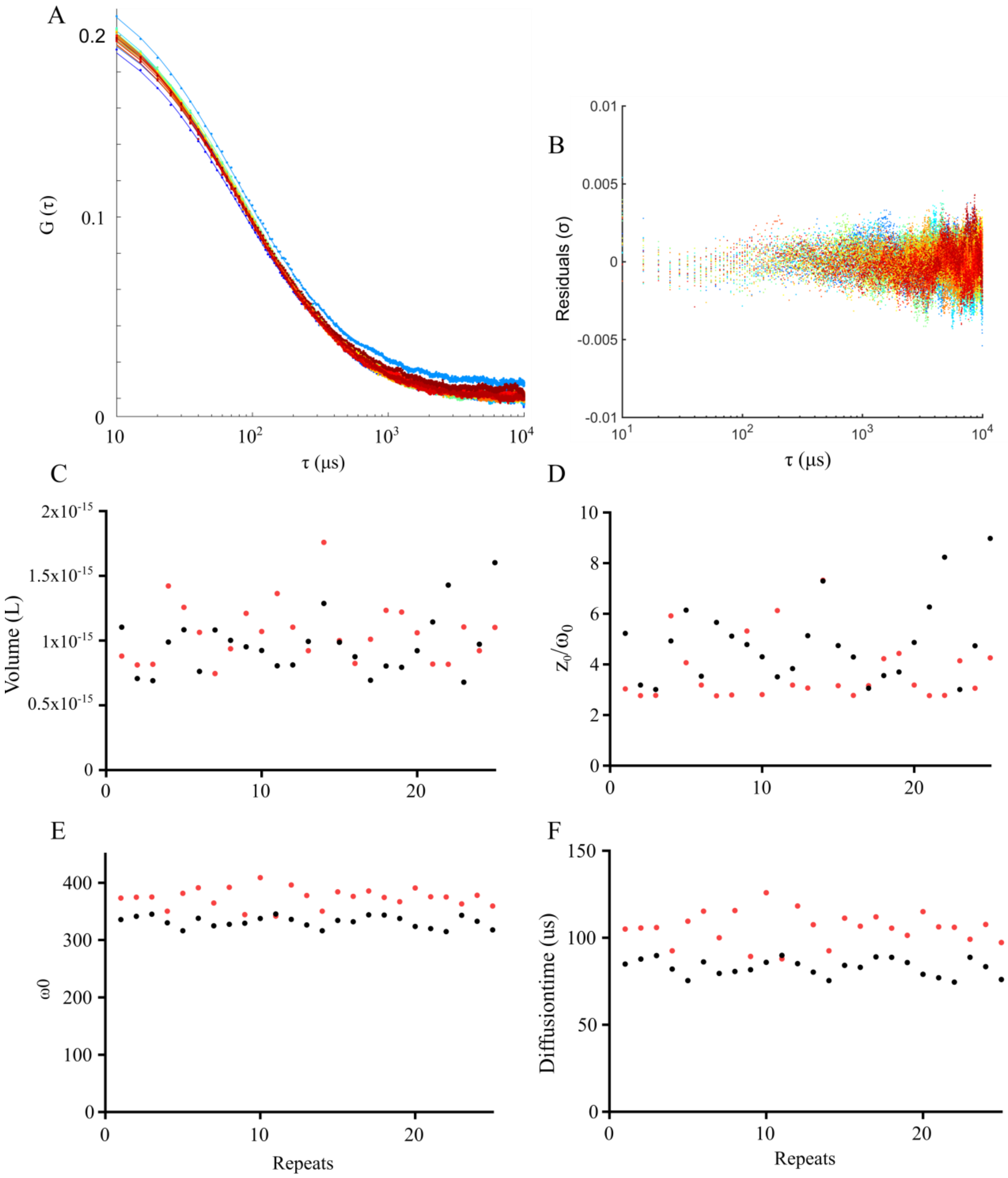


**Supplementary Figure 4 Repeated FCS measurements of free Alexa-568 dye performed on two separate instruments.**

(A) Autocorrelation curves of 20 nM Alexa-568 from 25 repeated 10 s measurements and fits to a one component diffusion model, allowing the physical parameters of the optical set-up to be calculated. (B) Residuals from fit in (A) (C) Confocal volume (L) for repeated measurements. Black, instrument A, red, instrument B.(D) Structure factor ($a = \frac{z_0}{\omega_0}$) for repeated measurements. Black, instrument A (a = 4.8 +/- 1.6), red, instrument B (a = 3.7 +/- 1.3). (D) Lateral radius ($\omega_0$) of confocal volume for repeated measurements. Black, instrument A ($\omega_0$ = 332 +/- 10 nm), red, instrument B ($\omega_0$ = 374 +/- 16 nm). (E) Diffusion times (μs) for repeated measurements. Black, instrument A $\tau_D$ = 83 +/- 5 μs), red, instrument B ($\tau_D$ = 106 +/- 9 μs).

Small differences in lateral radius between instruments mean that diffusion times do not fully overlap. However, the main variability in the volume calculation is from the structure factor leading to the same calculated volumes within error. Free dye is used as a standard to calculate focal volume after each re-alignment of the instrument.

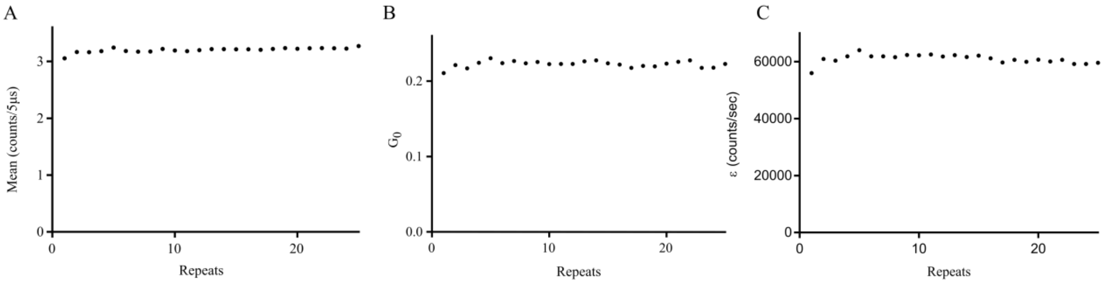


**Supplementary Figure 5 Repeated concentration and brightness measurements of free Alexa-568 dye**

(A) Mean intensity (B) Correlation amplitude, $G_0$ taken from FCS fit (C) Molecular brightness, ε. Experimental data taken from instrument A in Supp Fig 4.

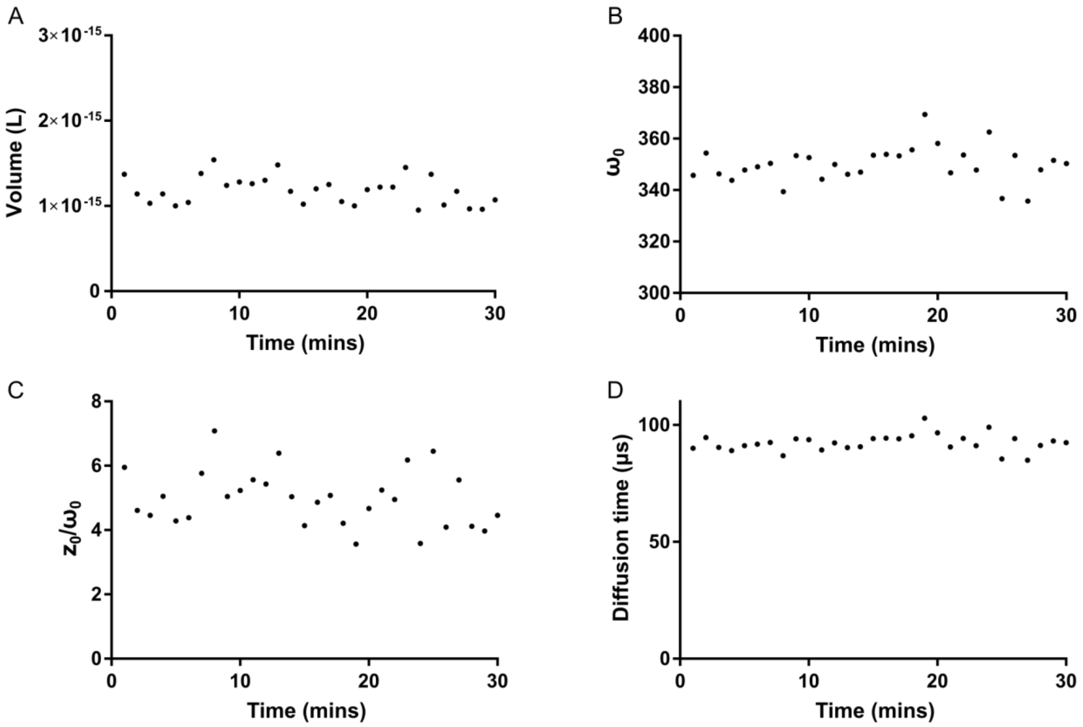


**Supplementary Figure 6 Repeated FCS measurements of free Alexa-568 dye performed across 30 mins.**

60s traces were obtained and the autocorrelation fit to a 3D Gaussian free diffusion model to obtain (A) Confocal volume (L) for repeated measurements. (B) Lateral radius ($\omega_0$) of confocal volume (C) Structure factor ($a = \frac{z_0}{\omega_0}$) (D) Diffusion times (µs).

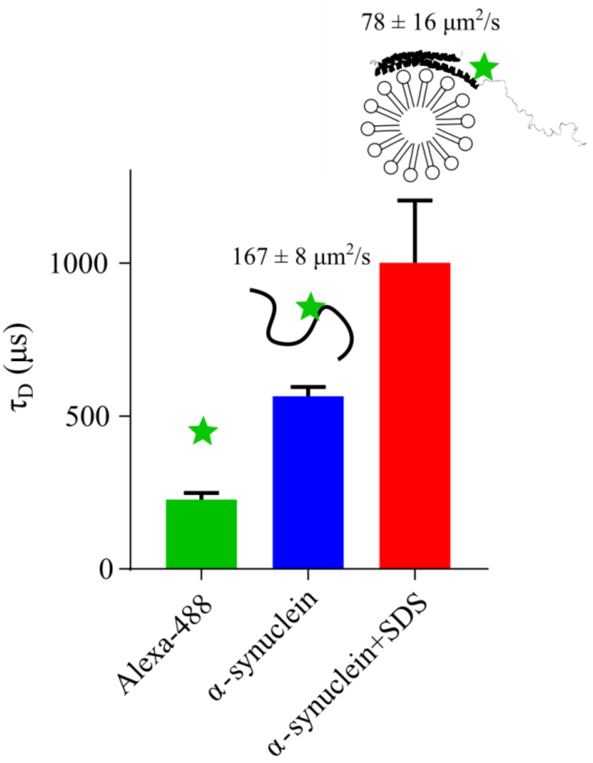


**Supplementary Figure 7** Characteristic diffusion times ($\tau_D$) of species measured in Fig 1D and calculated diffusion coefficients using Alexa-488 as a reference (414 μm$^2$/s). Data are mean + s.d. of 8 traces.

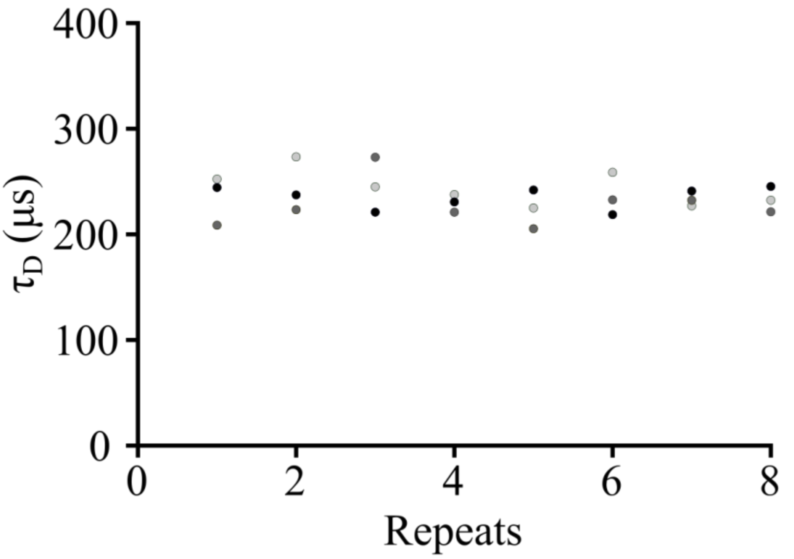


**Supplementary Figure 8** Diffusion times ($\tau_D$) of repeated measurements of free Alexa-488 measured at concentrations of 1 nM (green), 10 nM (yellow) and 100 nM (blue).

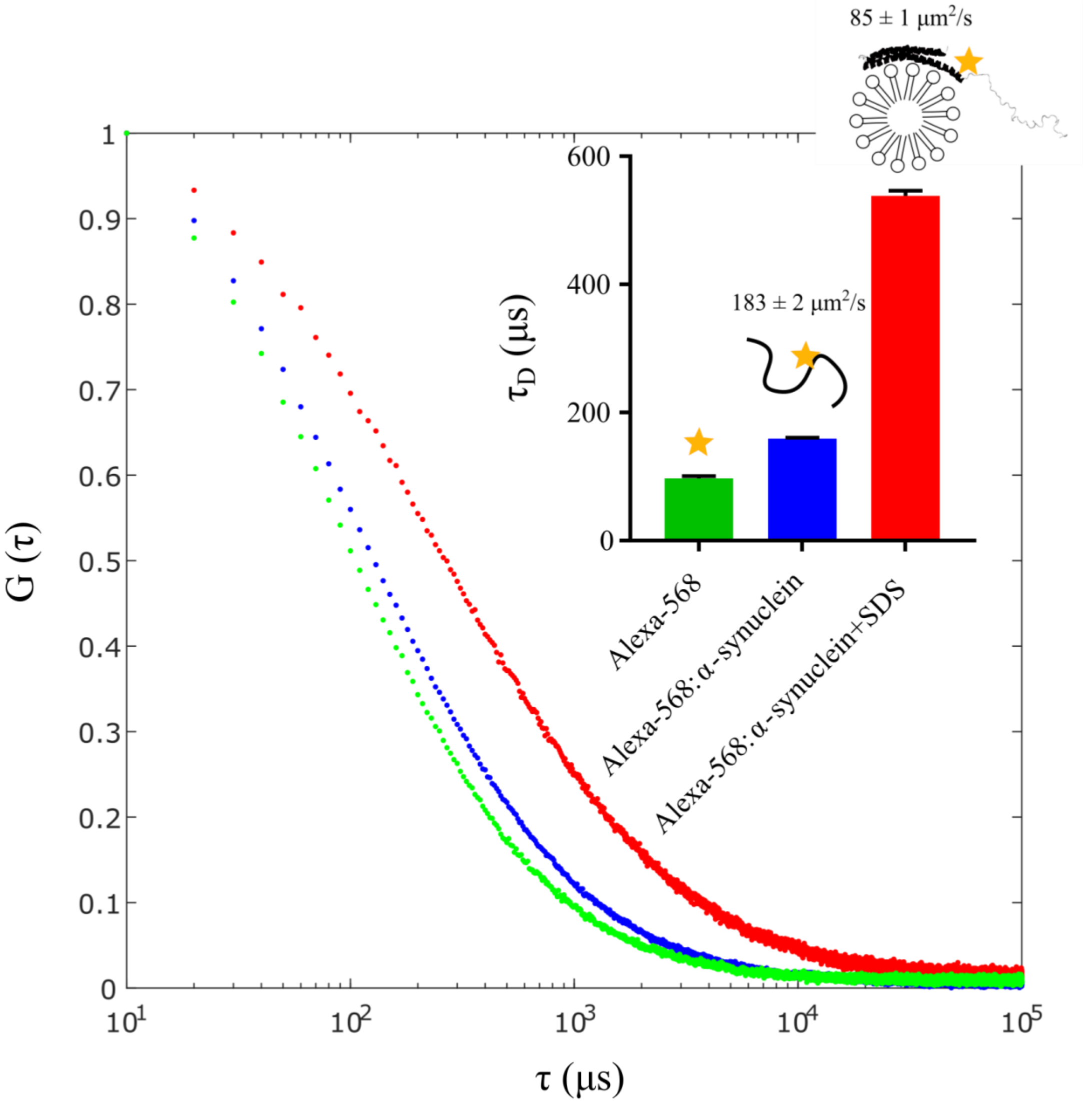


**Supplementary Figure 9** Fluorescence correlation spectroscopy (average of 8x10s traces acquired at 100kHz) of a fluorophore with a known diffusion coefficient (Alexa-568, 10 nM, green) and 10 nM Alexa-568 labelled α-synuclein in the absence (blue) and presence (red) of 10mM sodium dodecyl sulphate (SDS) micelles. Inset: Characteristic diffusion times ($\tau_D$) of species measured in Fig 1D and calculated diffusion coefficients using Alexa-568 as a reference. Data are mean + s.d. of 8 traces.

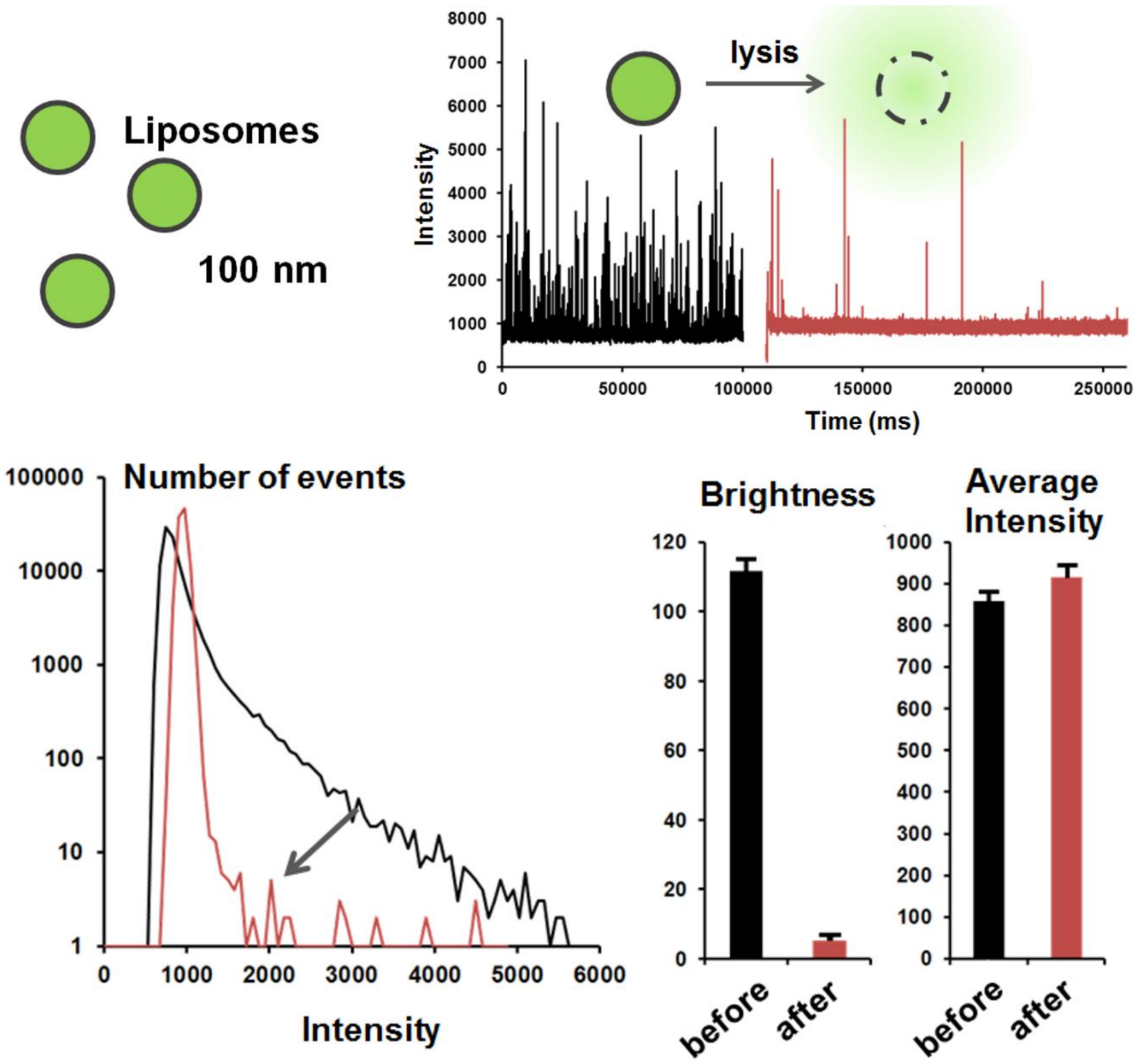


**Supplementary Figure 10** The AttoBright was tested on liposomes. Liposomes made of a 50:50 mixture of PC and PS (Avanti Polar Lipids) were extruded following the manufacturers protocol to a final size of 100 nm radius. They are filled with Alexa594, and the excess dye outside the liposomes was removed by gel filtration. **(top right):** here a proof-of-concept experiment was performed to demonstrate the applicability of the AttoBright to pore formation. In the first part of the fluorescent trace, liposomes are observed intact in buffer, and the detector records large bursts of intensity. After 100s, the laser was turned off, a 1% triton solution was mixed with the liposomes to mimic their leakage of fluorescence into solution. The laser was turned on again immediately after mixing, and the trace shows a slightly higher background, where few peaks remain. **(bottom left):** the analysis of distribution of intensity values before (black) and after (red) mixing show a clear transition in the number of liposomes measured. **(bottom right)** B values and mean intensity

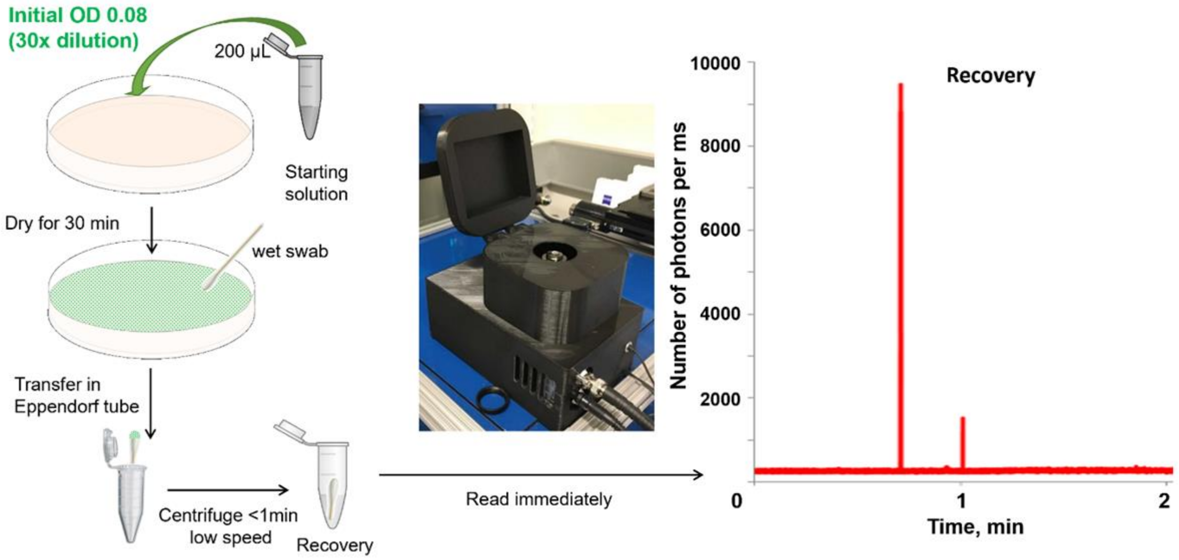


**Supplementary Figure 11** Bacterial detection on the AttoBright device. As a proof-of-concept for bacterial detection, we used *E. Coli* expressing mCherry as a test system. In a real test, specific antibodies would be used to mark the bacteria of interest. As shown on our previous experiments with protein fibrils, detecting objects above a constant background is simple in single-particle counting, and we tested the possibility of measuring the contamination of surfaces. As shown on the schematics, a low-density culture was spread on a plate, dried then recovered by touching the tip of a wet swab. The swab was transferred in a tube and the liquid was spun down and collected, and immediately measured on the AttoBright. The number of bacteria present in solution can be easily measured with single bacteria sensitivity.

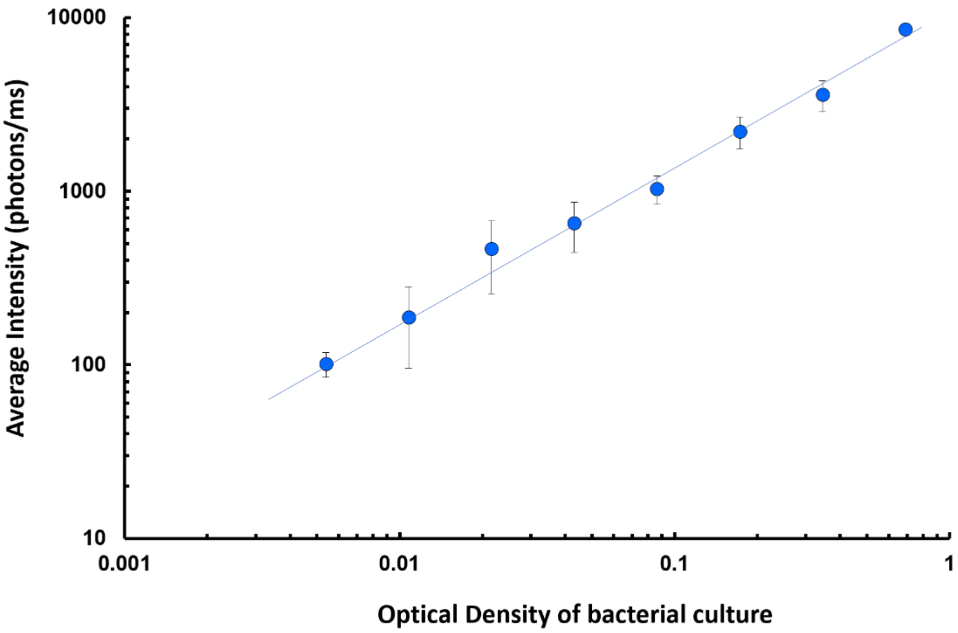


**Supplementary Figure 12** Measure of intensity of the trace as a function of O.D. for the culture of *E. Coli* expressing mCherry. This data show that the system behaves as expected and could be used to study slow-growing organisms.
