# Supplementary material for: Single-molecule detection on a portable 3D-printed microscope
Source: Nat Commun. 2019 Dec 11;10:5662. doi: 10.1038/s41467-019-13617-0 (PMC6906517; doi:10.1038/s41467-019-13617-0)
Supplement: Supplementary file 2 — Description of Additional Supplementary Files [file 41467_2019_13617_MOESM2_ESM.pdf]

Description of Additional Supplementary Files

File name: Supplementary Software

Description: The Supplementary Software file contains the 3D printing STL files, LabView code and a GUI for data acquisition, Matlab code and a GUI for data analysis as well as a parts list and user guide for assembly/acquisition and analysis.
