# Supplementary material for: Single-molecule detection on a portable 3D-printed microscope
Source: Nat Commun. 2019 Dec 11;10:5662. doi: 10.1038/s41467-019-13617-0 (PMC6906517; doi:10.1038/s41467-019-13617-0)
Supplement: Supplementary file 3 — Supplementary Software [file 41467_2019_13617_MOESM3_ESM.zip › AttoBright-1.0/index.html]

# AttoBright

## A 3D printed scaffold for single molecule spectroscopy

Gambin-Sierecki Lab1\*

1EMBL Australia Single Molecule Science node, UNSW, Australia

\*Permanent email: y.gambin@unsw.edu.au

To increase accessibility and reduce costs associated with single molecule detection capabilities, we designed a simplified optical path that can be housed in a single 3D printed block where all optical components are pre-aligned

Get it here

Original publication:

doi: TBC
