# Supplementary material for: Single-molecule detection on a portable 3D-printed microscope
Source: Nat Commun. 2019 Dec 11;10:5662. doi: 10.1038/s41467-019-13617-0 (PMC6906517; doi:10.1038/s41467-019-13617-0)
Supplement: Supplementary file 3 — Supplementary Software [file 41467_2019_13617_MOESM3_ESM.zip › AttoBright-1.0/Data Analysis Quick Guide.pdf]

AttoBright Analyser User Guide

James WP Brown

July 2019

1 Introduction

This app performs (1) basic fluorescence correlation spectroscopy (FCS) fitting, (2) the computation of statistical parameters and the calculation of a simplified molecular brightness parameter, and (3) the picking of peaks using a user-defined or inbuilt threshold. These computed parameters are saved as Microsoft Excel files. It will also plot raw data and histograms, as well as autocorrelated data + a fit, and these figures are saved as PDF documents.

2 Installation

2.1 Installation with MatLab Runtime

For Windows, download and install 'SMSInstaller_web.exe' which downloads a copy of MatLab Runtime if necessary

2.2 Installation with pre-installed MatLab software

Double click on the 'AttoBright Analyser.mlappinstal' file to install the package on the 'Apps' section of MatLab. Ensure that the current folder in MatLab is not the folder that contains the install file when the app is opened.

3 Data Input

Data must be input as a single column (one-colour experiments only), with each row being the number of photons detected in the integration time. This is the format of the data acquired using the LabView software accompanying the AttoBright paper.

4 Veryfrontpage

Initially, the user selects whether to analyse a single file or an entire folder of data. For the analysis of an entire folder there must be no files in the folder

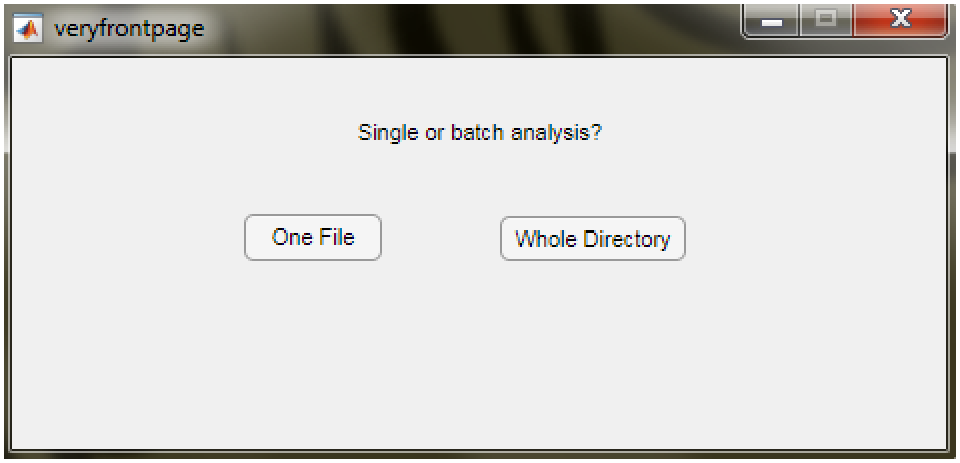

Figure 1: Single or Multi GUI

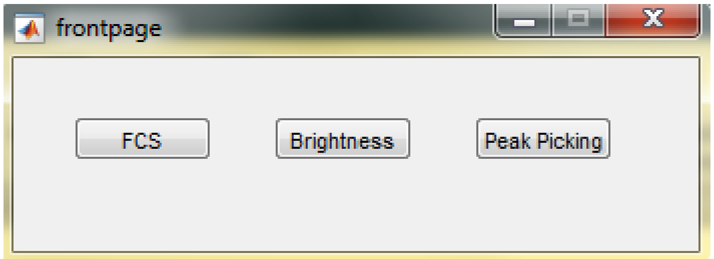

Figure 2: Frontpage GUI

other than the raw data to be analysed. The remainder of the manual will refer to the single file analysis, the bulk analysis is identical except a directory is chosen rather than a file, and results are saved as a single Excel file with data organised by filename.

5 Frontpage

The user selects one of three general options for the analysis method which is to be performed.

(1) FCS
(2) Brightness
(3) Peak Picking

Each option opens another window.

6 FCS

For FCS fitting, a non-linear least squares algorithm is used to fit one of four equations (depending on number of species and triplet state kinetics) for the diffusion of a molecule through a 3D Gaussian volume.
The autocorrelation function is calculated before fitting to either:
(1) A one component diffusion model with triplet state correction

$$G_\tau = (1 + (\tau_p/(1-\tau_p)) * e^{(-x/\tau_e)}) \\ * G_0/((1 + (x/\tau_D)) * (1 + a^{-2} * (x/\tau_D))^{1/2}) + G_\infty \quad (1)$$

(2) A one component diffusion model (no triplet state correction)

$$G_\tau = G_0/((1 + (x/\tau_D)) * (1 + a^{-2} * (x/\tau_D))^{1/2}) + G_\infty \quad (2)$$

(3) A two component diffusion model with triplet state correction

$$G_\tau = (1 + (\tau_p/(1-\tau_p)) * e^{(-x/\tau_e)}) \\ * G_0 * (f/(1 + (x/\tau_D)) * 1/(1 + a^{-2} * (x/\tau_D))^{1/2} \\ + ((1-f)/(1 + x/\tau_2)) * 1/(1 + a^{-2} * (x/\tau_2))^{1/2}) + G_\infty \quad (3)$$

(4) A two component diffusion model (no triplet state correction)

$$G_\tau = G_0 * (f/(1 + (x/\tau_D)) * 1/(1 + a^{-2} * (x/\tau_D))^{1/2} \\ + ((1-f)/(1 + x/\tau_2)) * 1/(1 + a^{-2} * (x/\tau_2))^{1/2}) + G_\infty \quad (4)$$

Where G_τ is the autocorrelated data, G_0 is the correlation at $\tau = 0$, τ_p is the fraction of fluorophore in the triplet state, τ_e is the corresponding triplet state relaxation time, τ_D is the characteristic diffusion time of the fluorescent species, Where $a = z_0/\omega_0$ is the structure factor (where z_0 and ω_0 are the e^{-2} radii in the lateral and perpendicular direction relative to the optic axis, respectively) , and G_∞ is the value of the autocorrelation as $\tau \to \infty$.

Results of the fit, as well as the root mean square error are saved automatically as [filename,modelused] (e.g. testFCSonecomp) and a figure with the autocorrelated data and the fit saved in pdf format in a /Figures folder under the same name.

Units are in μs based on a 100kHz acquisition frequency. These must be corrected for different acquisition frequencies.

7 Brightness

Under this option, basic statistical parameters (mean, standard deviation, mode) are calculated along with calculations of the B parameter (σ^2/μ, Gambin et

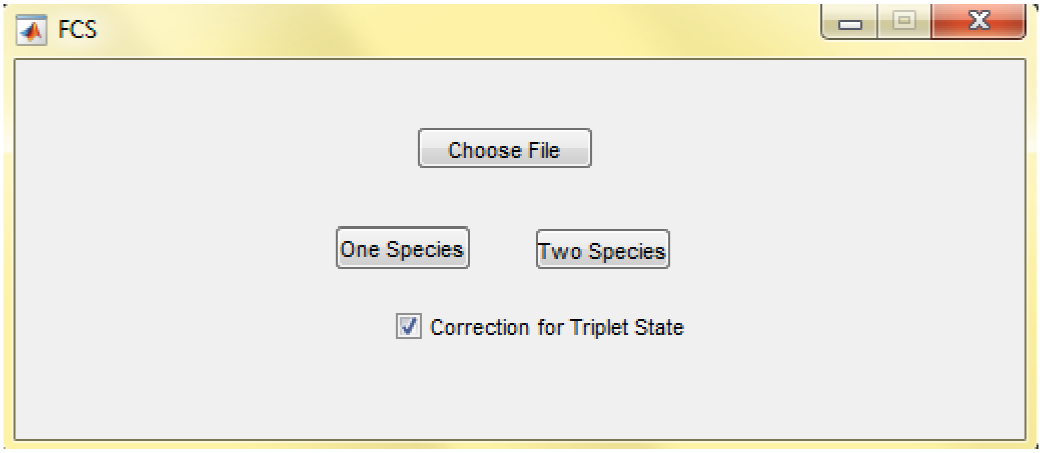

Figure 3: FCS GUI

al, IJMS 2016) and Brightness ((σ^2/μ)-1) according to (Digman et al, Biophys J 2008). Results are saved as a Microsoft Excel file with the name [filename,brightness] (e.g. testbrightness).

8 Peak Picking

This option performs the picking of individual fluorescent events above a threshold, which allows the deconvolution of number and intensity data of intrinsically or extrinsically labelled samples. Thresholding can be performed either manually (e.g. with reference to a control trace) or by using an inbuilt threshold of mean + 3 standard deviations.

In this analysis, once the fluorescence intensity is above threshold, all subsequent time-points until the intensity reduces below threshold are counted as one peak and the parameters that are output are the peak number, the integrated intensity of all peaks, the average maximum peak intensity, the total number of timepoints above threshold, as well as the mean and B parameter for the entire trace. Results are saved as a Microsoft Excel file with the name [filename,peakpick] or [filename,peakpickuserthresh] depending on threshold used.

The plot button can also be used to plot the raw data and save in pdf format in a /Figures folder under the raw data file name.

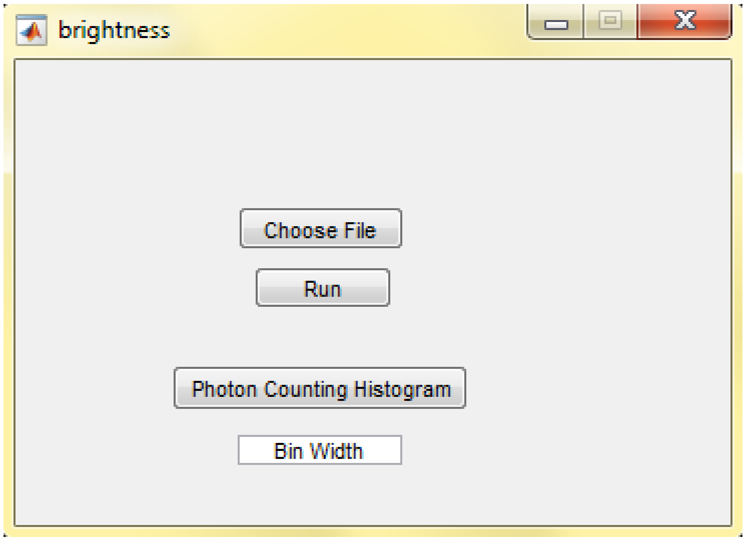

Figure 4: Brightness GUI

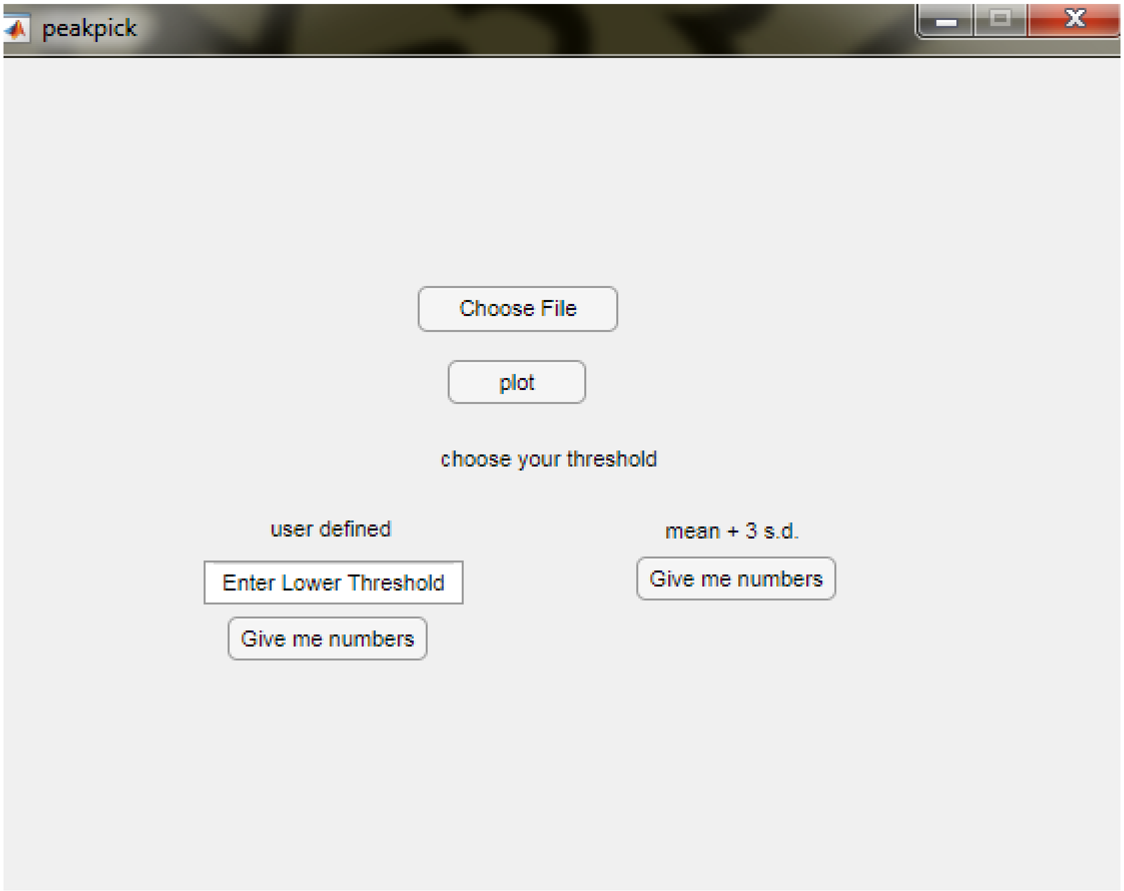

Figure 5: Peak Picking GUI
