# Supplementary material for: Single-molecule detection on a portable 3D-printed microscope
Source: Nat Commun. 2019 Dec 11;10:5662. doi: 10.1038/s41467-019-13617-0 (PMC6906517; doi:10.1038/s41467-019-13617-0)
Supplement: Supplementary file 3 — Supplementary Software [file 41467_2019_13617_MOESM3_ESM.zip › AttoBright-1.0/Data Acquisition LabView programs/Data Acquisition Quick Guide.docx]

**Data Acquisition Quick Guide**

1. Adjusting the height of the NanoBright Objective:
2. Connecting the NanoBright to the power source and computer.
3. Previewing signal and aligning the NanoBright
4. Acquiring signal on the NanoBright
5. **Adjusting the height of the NanoBright Objective:**

The height adjustment of the objective (focussing) is very critical for proper data acquisition on the NanoBright. If not set at the correct height, you would not acquire maximum signal from your sample.

- NanoBright is fitted with holder for the objective, which is screw tightened into the plastic scaffold.


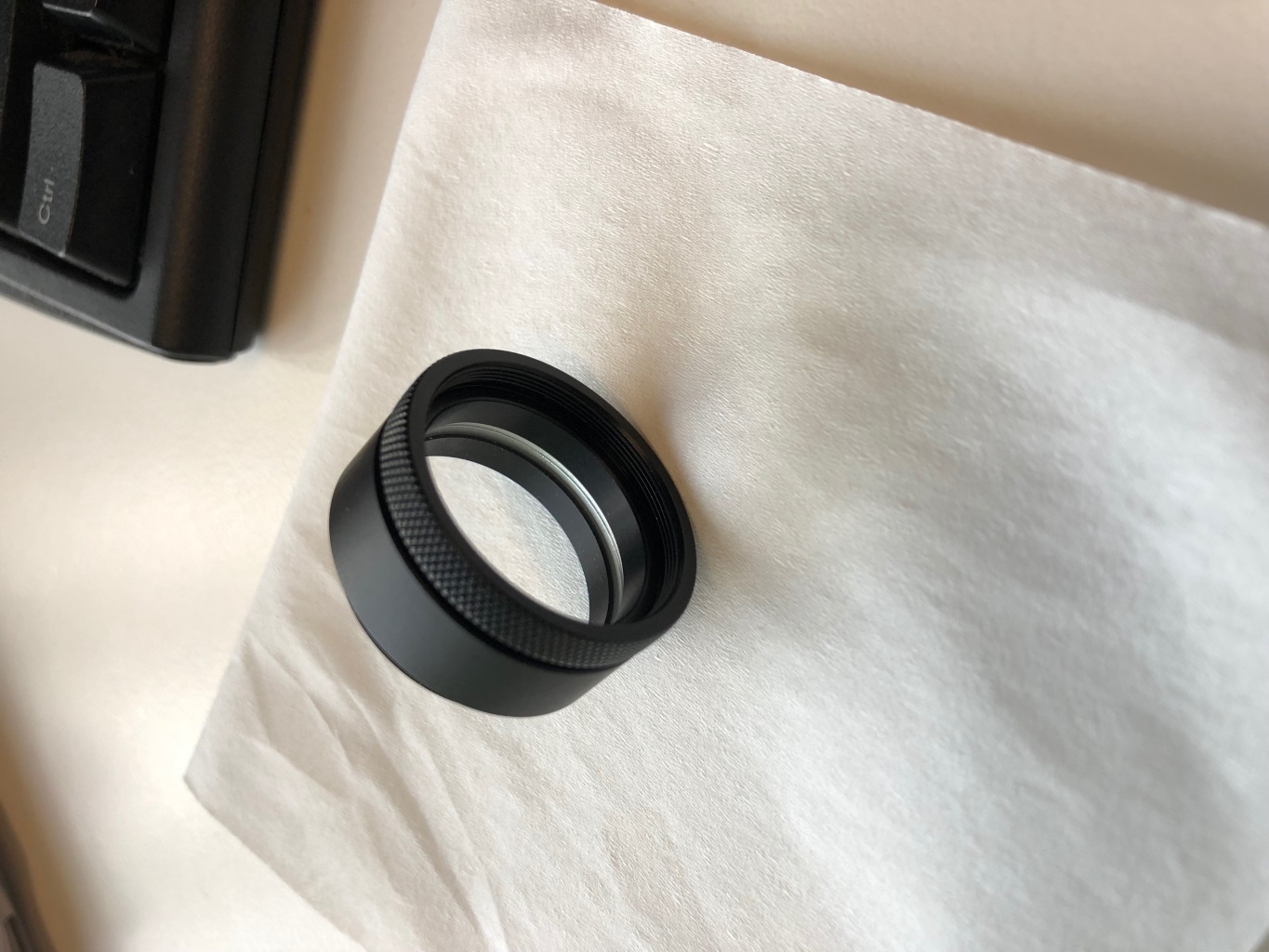


-
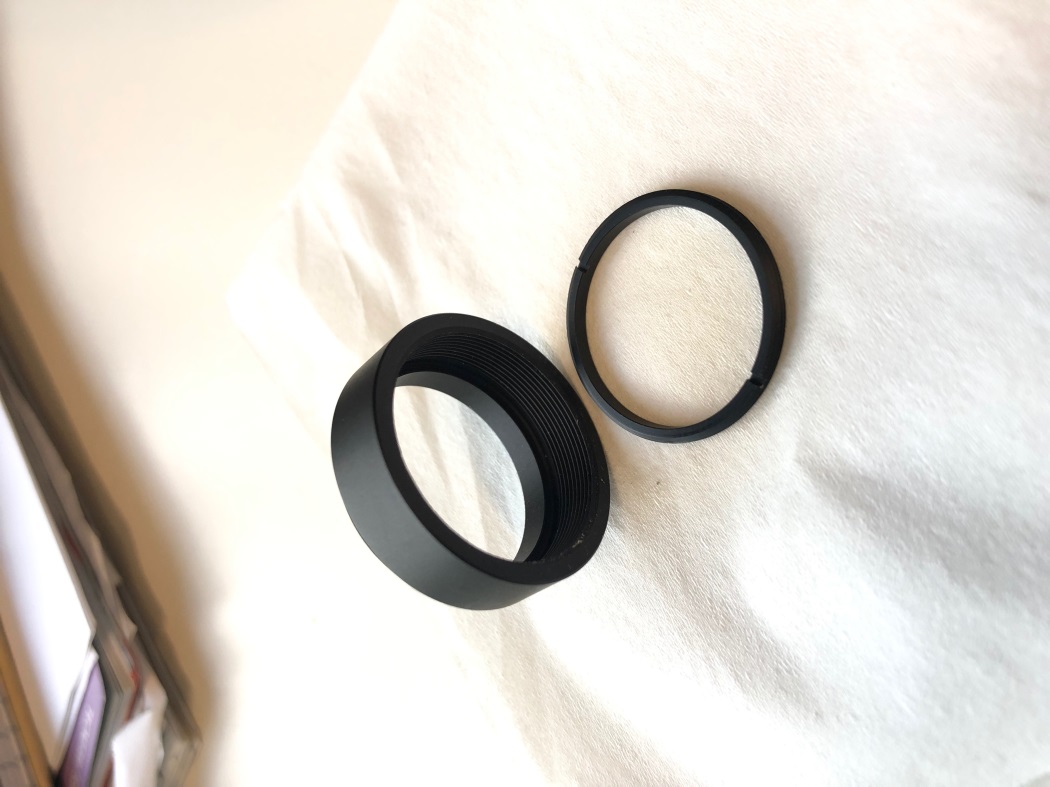
This holder has threads on the inside which are used to screw the objective in. In addition to this, the holder has another ring inside it, which is to be used to change the height of the objective upwards or downwards.
- To set the height of the objective, screw the inner ring to the holder and then screw the objective tight on to the holder. For running any experiments, you need to make sure that the objective is screwed in tight with the holder.


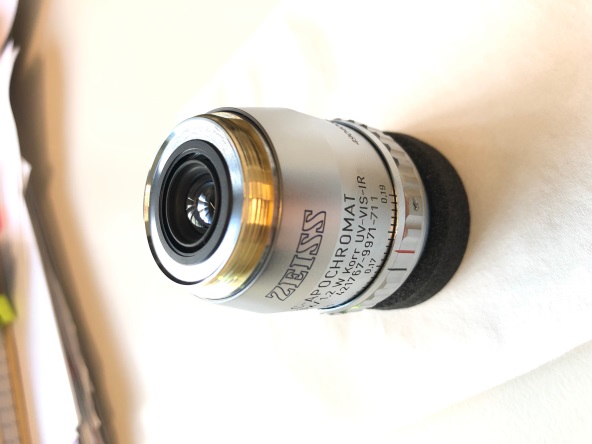

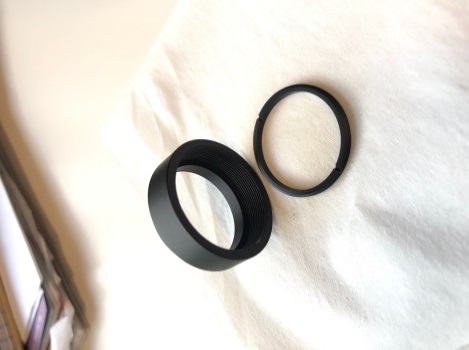

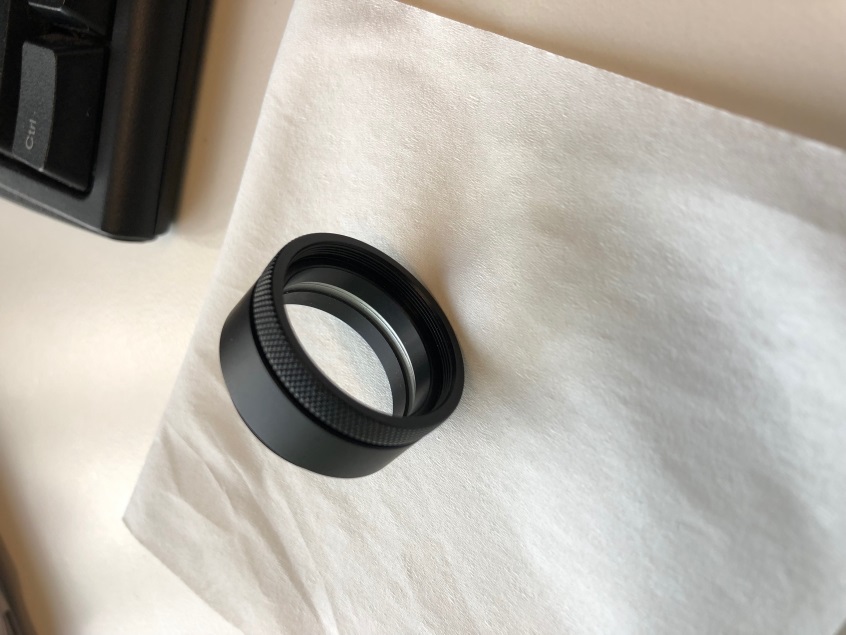


- To check if the objective is at the correct height, screw the objective and the inner ring to the holder.
- Once all screwed in, put the objective holder cover back on, and then slide a glass coverslip over the objective.
- If the objective is too low, you’ll see a gap between objective and the glass coverslip and if the objective is too high, the glass coverslip will get lifted up.
- Once you adjust the height of the objective, add 100µL of nuclease free (ultra-pure) water on the objective and place the glass coverslip on it.
- If the height of the objective is optimum, you’ll observe that the glass is in full contact with the water and the objective and you cannot press down on the glass anymore.

1. **Connecting the NanoBright to the power source and computer**

Once the NanoBright is fully assembled and all parts are screwed in, please follow the following steps to connect the NanoBright to the power source and computer.

Connecting the Laser power:

-
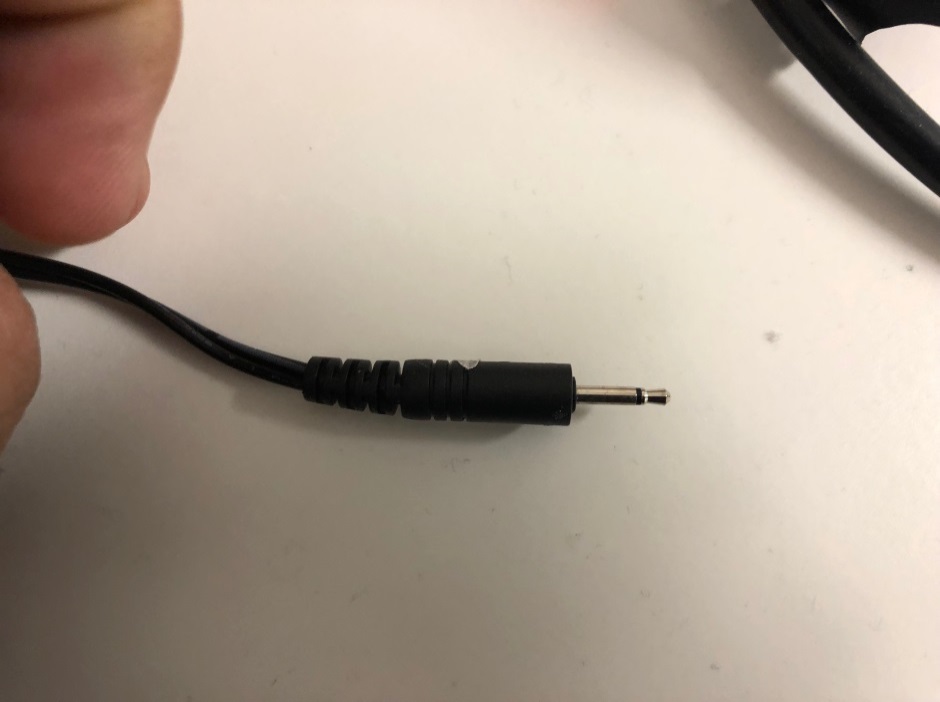

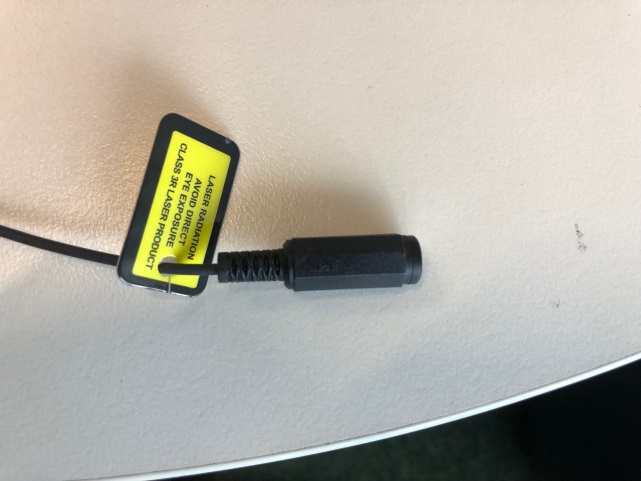
To power the laser, connect the following two ports and plug the power cord to the mains power supply.
-
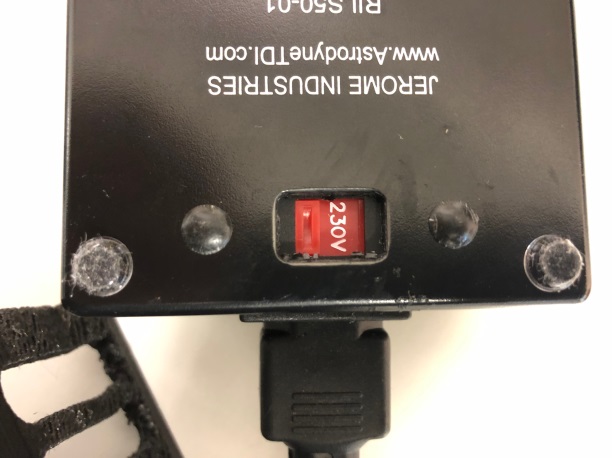
Ensure that the voltage is set to correct settings suited to your mains power.

Connecting the Detector to DAQ card:

-
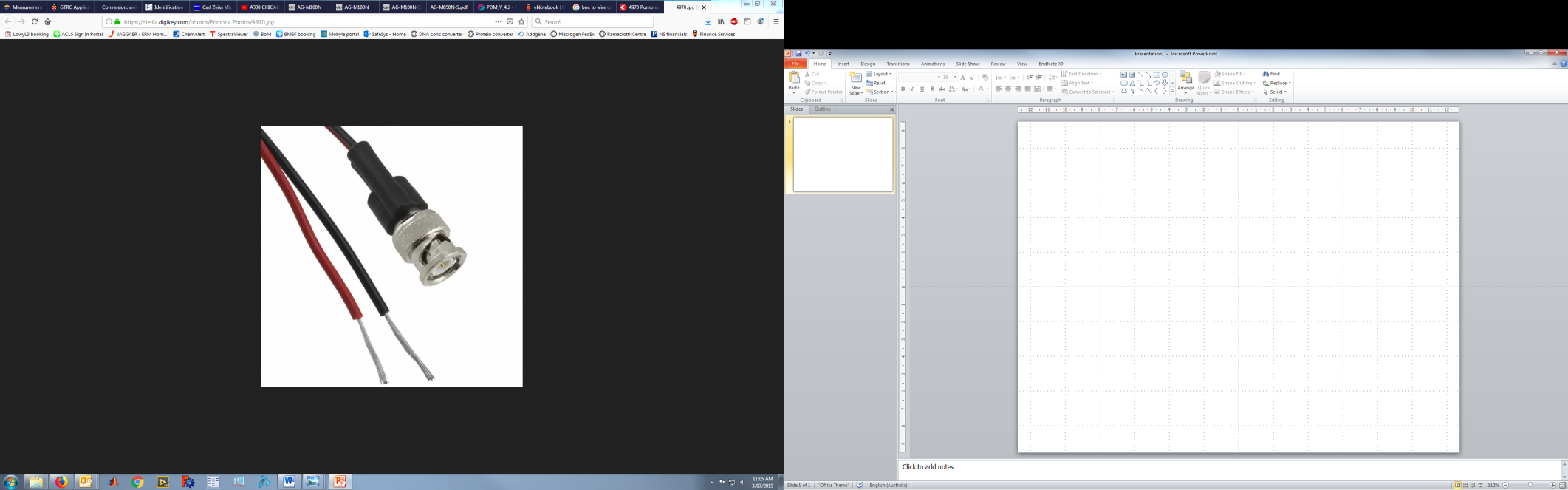
To connect the Detector to DAQ card, you need the BNC to wire connectors
- Connect the black cable of the BNC to the GND of the DAQ card and red to COIN.
- Once secured, connect the other end of the BNC to the out port of the detector.

(for clarity, see the images below).


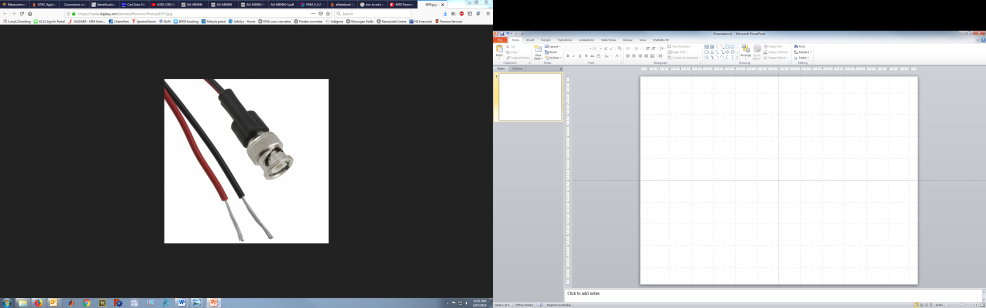

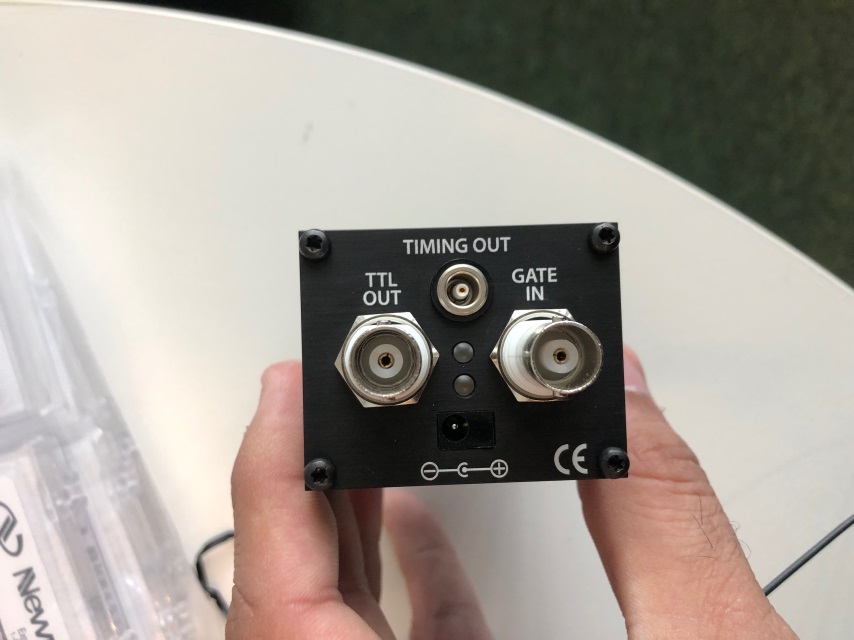

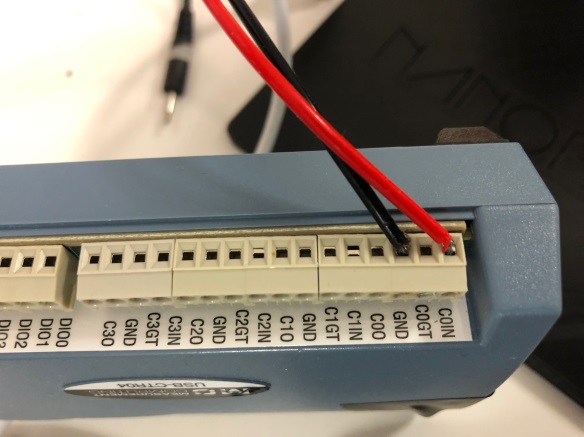


Detector

BNC to wire connector

DAQ Card

- Once the detector is connected to the DAQ card, connect the power supply to the detector which then connects to the mains power supply.

Connecting the DAQ to the computer

- Use the USB cable provided with the DAQ card to connect the DAQ card to the USB port of the PC.
- Upon hardwiring the DAQ card, open the software ‘InstaCAL’ on your PC. This should automatically connect the DAQ card to the PC board.


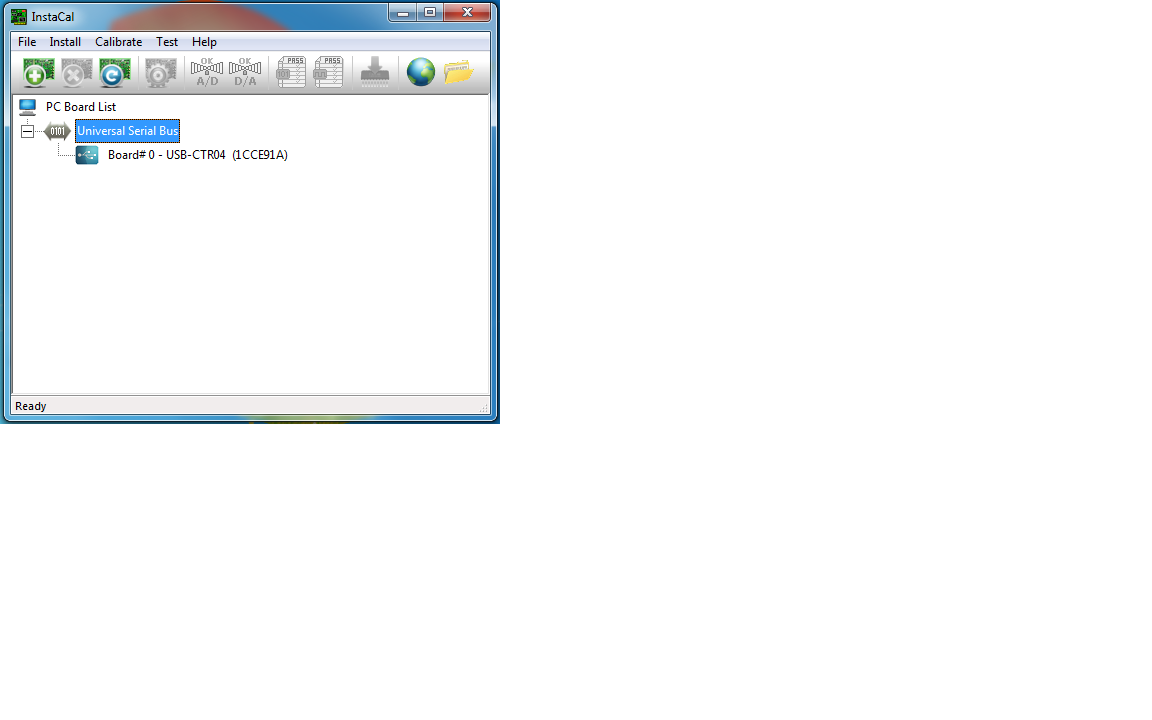


Connecting the Piezo Controller to the computer

-
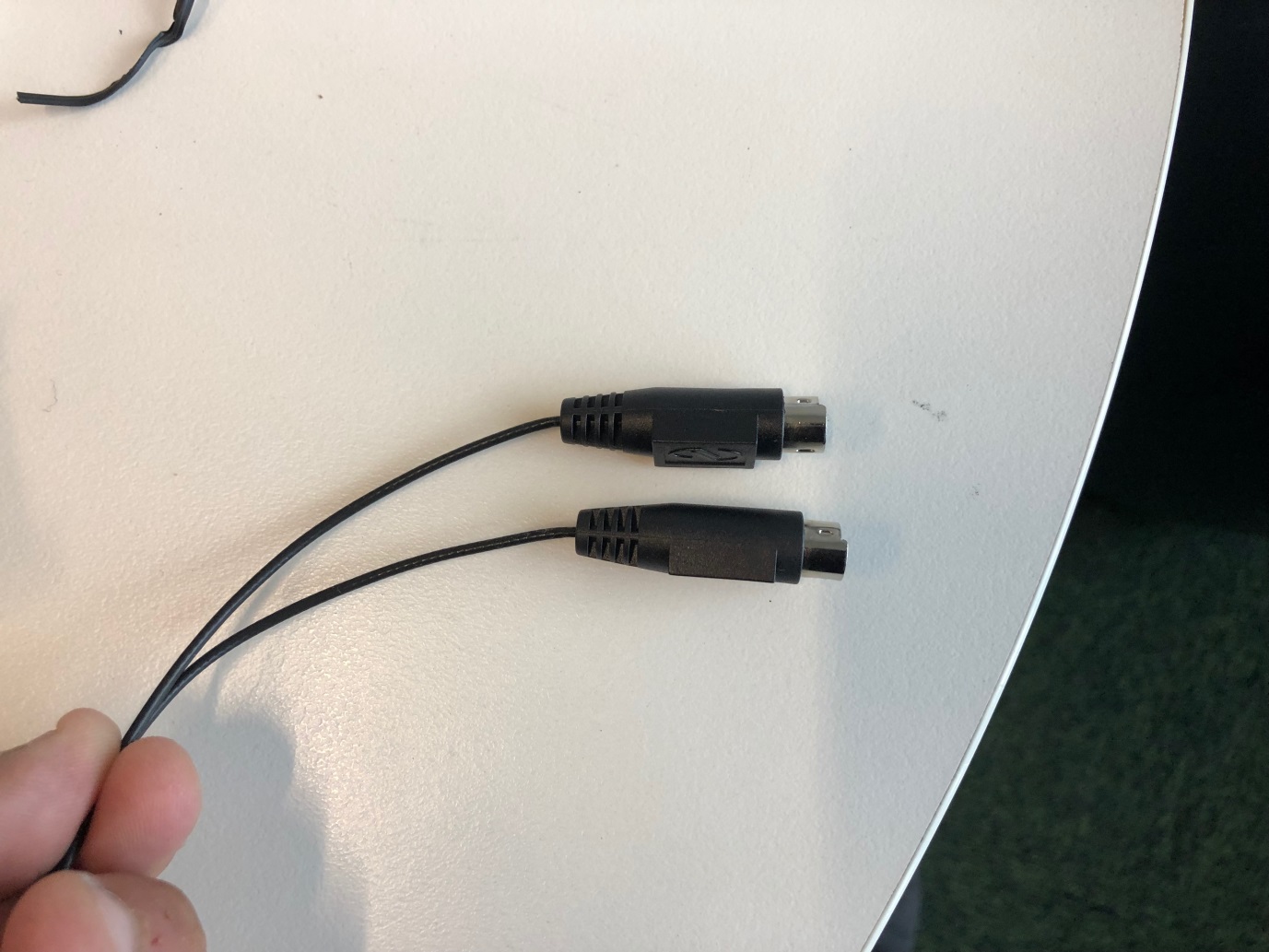
From your NanoBright, you should see two cables (like in the picture) coming out of the NanoBright.
- Connect these cables to the piezo controller and connect the piezo controller to the computer using the USB cable provided with the piezo controller.


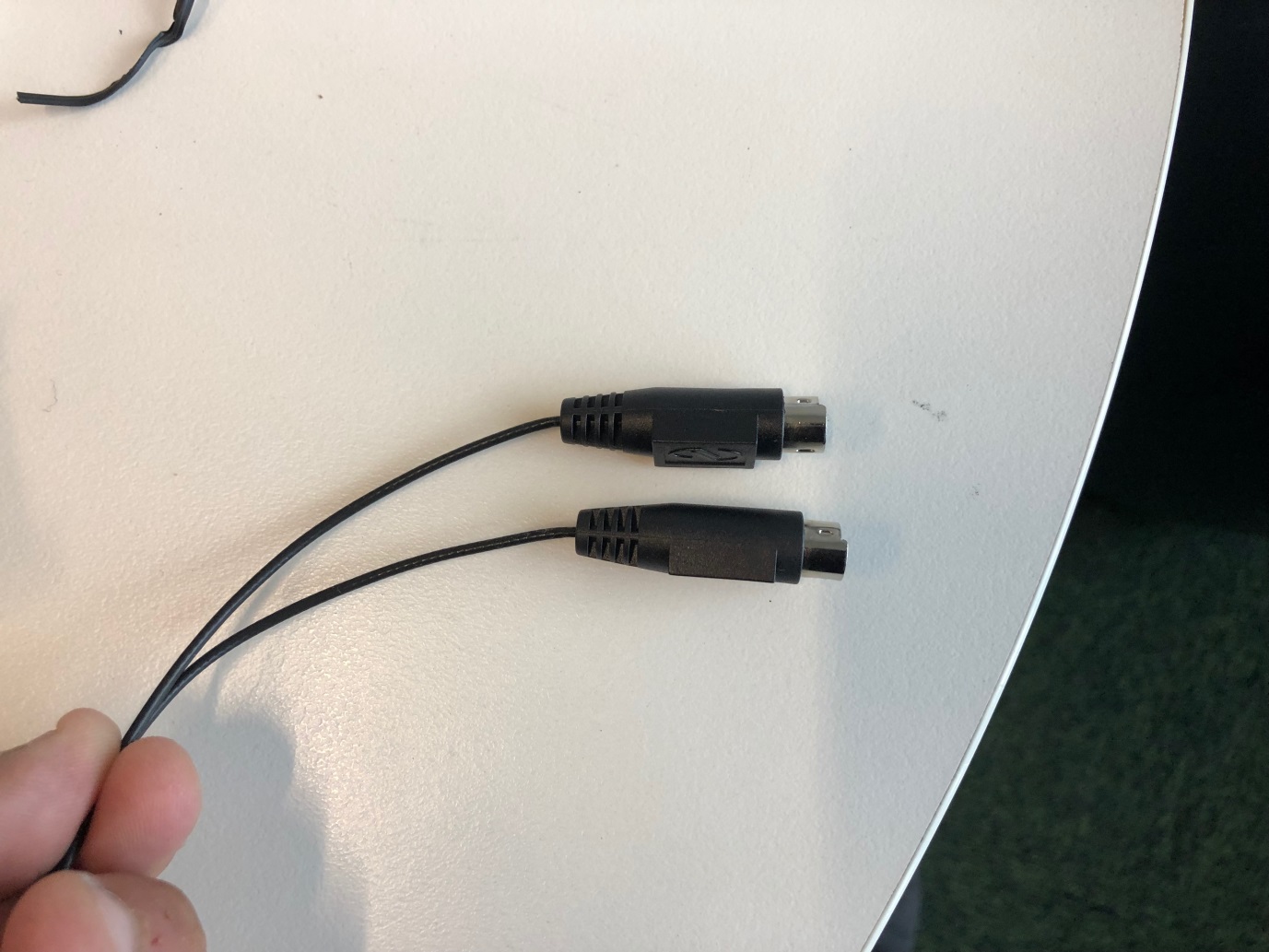


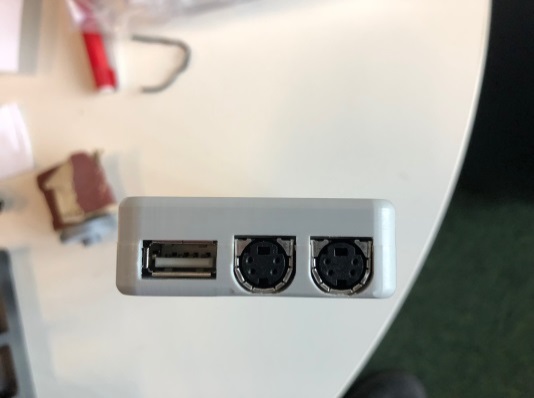


- It is important to know which USB port on the computer you are connecting the piezo controller. To find the exact port number, go to ‘Device Manger’ on your computer and look for ‘Aiglis’. Under the properties of this device, you’ll find what port number it is connected to.
- This port number will be fed in to the acquisition software which would allow you to use the self-alignment functionality of the acquisition software.

1. **Previewing signal and aligning the NanoBright**

*(Only for previewing sample and not acquiring)*

The previewing routine is useful to judge the signal before starting a full measurement and also to align the NanoBright.

-
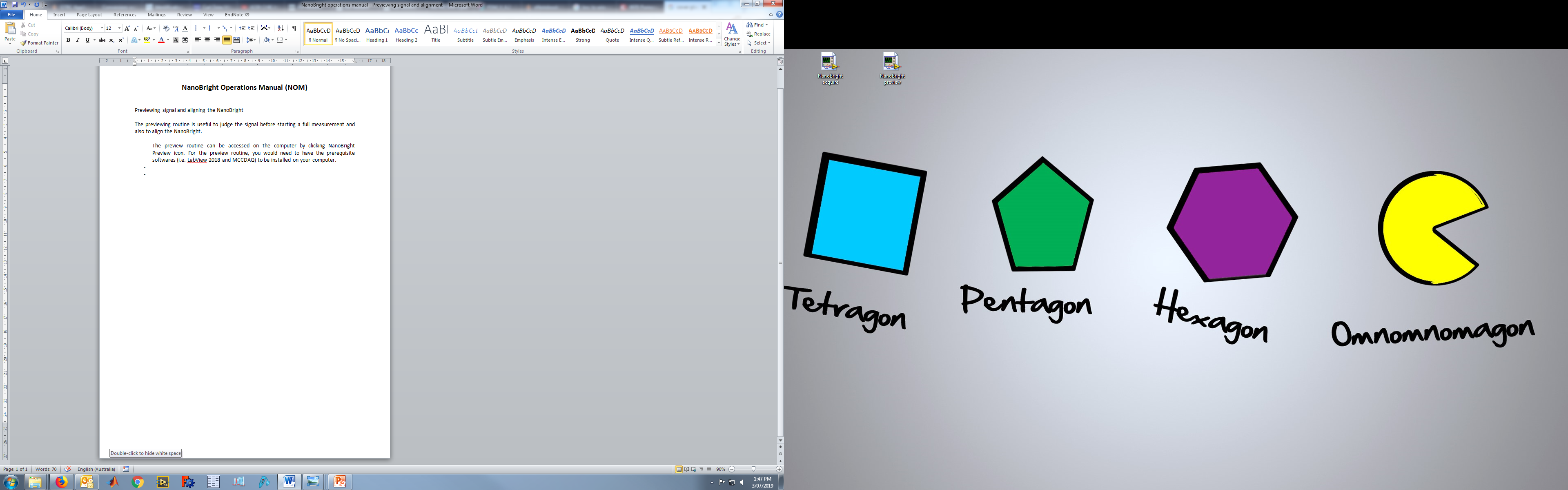
The preview routine can be accessed on the computer by clicking NanoBright Preview icon. For the preview routine, you would need to have the prerequisite softwares (i.e. LabView 2018 and MCCDAQ) to be installed on your computer.
- Before you start previewing your signal, please make sure that you have alignment sample loaded on the plate and plate is in the plate is in contact with the objective (via water / oil depending on the type of objective used) with the well centred around the laser.
-
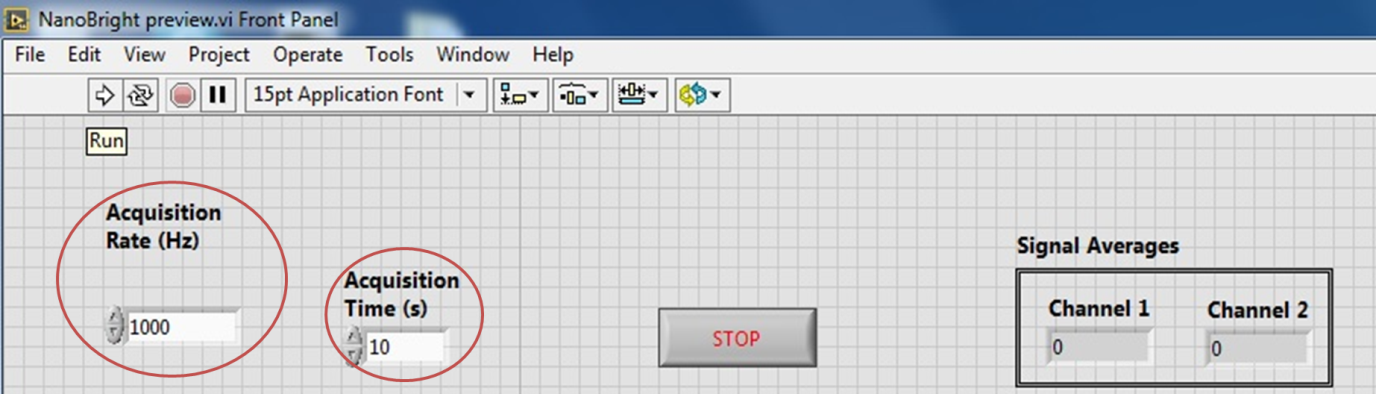
Before you start the preview, you need to choose two settings, (1) the time you want to preview the signal and (2) Acquisition rate (Hz) at which you want to preview the sample at.
- To start the routine, click on run icon ( ) and this would start the preview.
- While previewing your sample, you can use the piezo controller to align your sample. With sample previewing on, use either the left / right controls to move the mirror attached to the piezo to get optimum signal.
- On the piezo controller you can use the coarse adjustments (medium / ) or fine adjustment (small / ) to move the mirror to get optimum signal.
- Once you have reached to the optimum, you’ll notice that upon further movement to one or both direction (i.e up or down) your signal reduces.
- For a complete alignment, make sure you move the coarse and the fine adjustment in both the axis of the piezo to reach highest possible signal.
- Importantly, since the active area of the detector acts as the pinhole for the instrument, the position of the detector within the housing must also be optimised for complete alignment of the confocal spectrometer. The detector has space to move back and forward within the housing and can be carefully adjusted by hand.


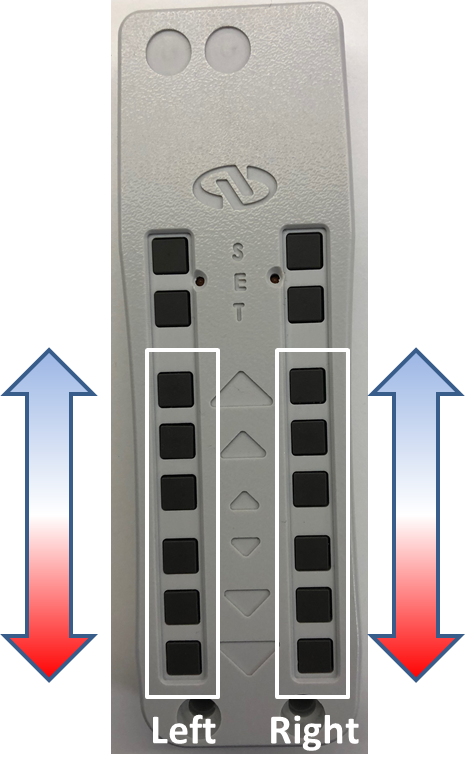


1. **Acquiring signal on NanoBright**

The acquire routine is to set the data acquisition parameters on the NanoBright and to acquire data for analysis.

- To start the acquire routine, double click on the NanoBright acquire. Before you do so, please make sure that you have LabView 2018 and MCCDAQ installed on your computer.


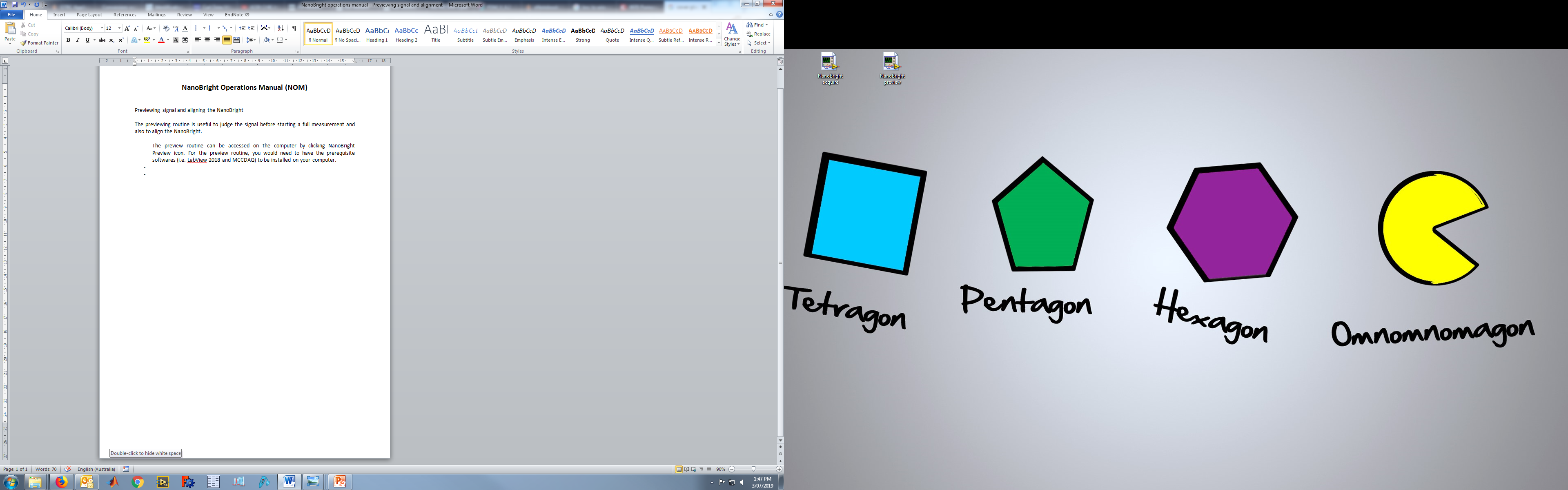


- Before you start the data acquisition, please make sure that you have aligned the NanoBright (either manually or using the self-align algorithm) and you have sample in your well and the well is centred on the laser.
-
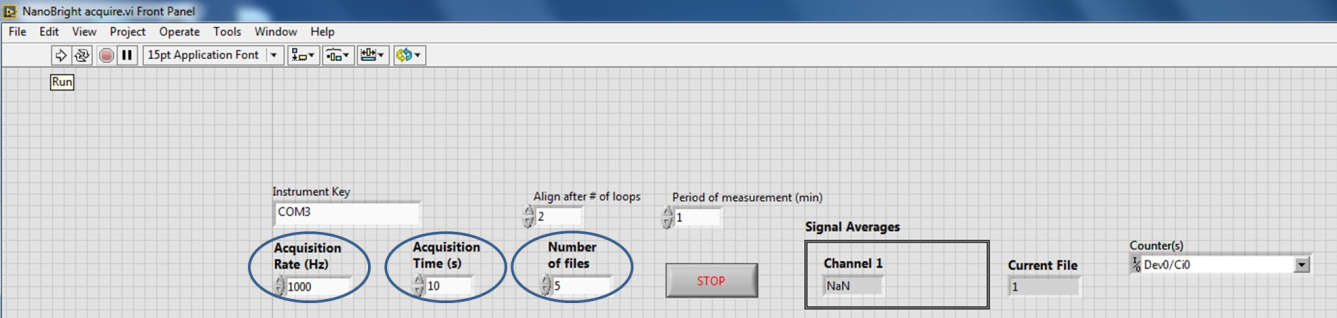
For data acquisition, you can modify three parameters, (1) the Acquisition rate, (2) Acquisition time and (3) Number of files.
- You can program the system to read multiple files one after the other for the same well. However, the transition from one well to the other has to be done manually by moving the plate so that the new well is centred on the laser
- The program gives you the flexibility of acquiring the sample at different acquisition rates depending on what type of data do you want to collect and what result you want to achieve.
-
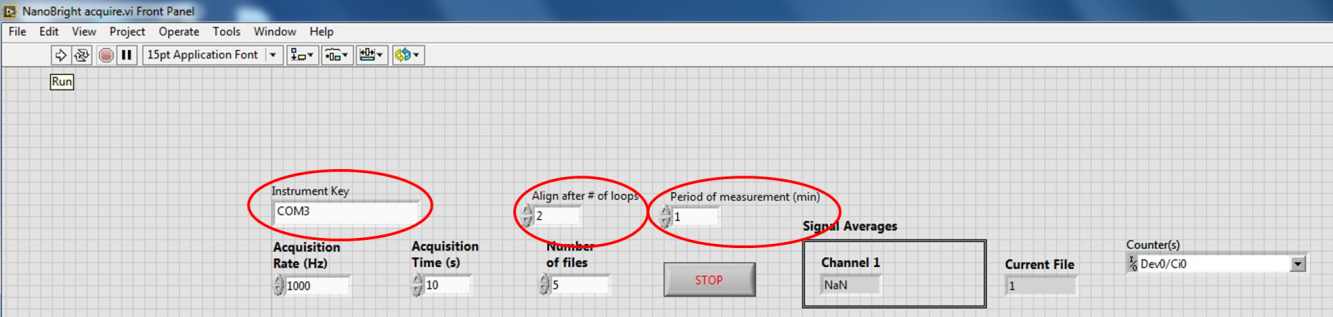
Another feature that you can choose to set for this measurement is the self-alignment. While programming the acquisition, you can ask program the machine to self-align after ‘x’ loops or after ‘x’ minutes of measurement.
- For the self-alignment to work, you need to make sure the ‘instrument key’ number is correctly entered. This number corresponds to the USB port number to which the piezo controller has been connected (refer to connecting the piezo controller under connecting the NanoBright to computer and power source document).
- To start the measurement, click run ( ) which will prompt you to choose the folder and the filename in which you would like to save your data.
